# Elevated CO_2_-Induced Responses in Stomata Require ABA and ABA Signaling

**DOI:** 10.1016/j.cub.2015.09.013

**Published:** 2015-10-19

**Authors:** Caspar Chater, Kai Peng, Mahsa Movahedi, Jessica A. Dunn, Heather J. Walker, Yun-Kuan Liang, Deirdre H. McLachlan, Stuart Casson, Jean Charles Isner, Ian Wilson, Steven J. Neill, Rainer Hedrich, Julie E. Gray, Alistair M. Hetherington

**Affiliations:** 1 Department of Molecular Biology and Biotechnology, University of Sheffield, Firth Court, Western Bank, Sheffield S10 2TN, UK; 2 School of Biological Sciences, Life Sciences Building, University of Bristol, Woodland Road, 24 Tyndall Avenue, Bristol BS8 1TQ, UK; 3 Department of Animal and Plant Sciences, University of Sheffield, Alfred Denny Building, Western Bank, Sheffield S10 2TN, UK; 4 State Key Laboratory of Hybrid Rice, College of Life Sciences, Wuhan University, Wuhan 430072, China; 5 Faculty of Health and Life Sciences, University of the West of England, Bristol Frenchay Campus, Coldharbour Lane, Bristol BS16 1QY, UK; 6 Institute for Molecular Plant Physiology and Biophysics, University of Würzburg, 97082 Würzburg, Germany

**Keywords:** guard cells, stomata, ABA receptors, [CO_2_] signaling, ABA signaling, NADPH oxidases, *Rboh* genes, signaling convergence, ROS, stomatal closure, stomatal density

## Abstract

An integral part of global environment change is an increase in the atmospheric concentration of CO_2_ ([CO_2_]) [1]. Increased [CO_2_] reduces leaf stomatal apertures and density of stomata that plays out as reductions in evapotranspiration [2–4]. Surprisingly, given the importance of transpiration to the control of terrestrial water fluxes [5] and plant nutrient acquisition [6], we know comparatively little about the molecular components involved in the intracellular signaling pathways by which [CO_2_] controls stomatal development and function [7]. Here, we report that elevated [CO_2_]-induced closure and reductions in stomatal density require the generation of reactive oxygen species (ROS), thereby adding a new common element to these signaling pathways. We also show that the PYR/RCAR family of ABA receptors [8, 9] and ABA itself are required in both responses. Using genetic approaches, we show that ABA in guard cells or their precursors is sufficient to mediate the [CO_2_]-induced stomatal density response. Taken together, our results suggest that stomatal responses to increased [CO_2_] operate through the intermediacy of ABA. In the case of [CO_2_]-induced reductions in stomatal aperture, this occurs by accessing the guard cell ABA signaling pathway. In both [CO_2_]-mediated responses, our data are consistent with a mechanism in which ABA increases the sensitivity of the system to [CO_2_] but could also be explained by requirement for a CO_2_-induced increase in ABA biosynthesis specifically in the guard cell lineage. Furthermore, the dependency of stomatal [CO_2_] signaling on ABA suggests that the ABA pathway is, in evolutionary terms, likely to be ancestral.

## Highlights

• CO_2_-induced stomatal closure and density reduction require reactive oxygen species
• CO_2_-induced stomatal closure and density reduction require ABA and ABA receptors
• Guard cell/precursor ABA is sufficient to mediate closure and density reduction
• Stomatal CO_2_ responses operating via ABA explains overlap between these pathways

## Graphical Abstract

**Figure:**
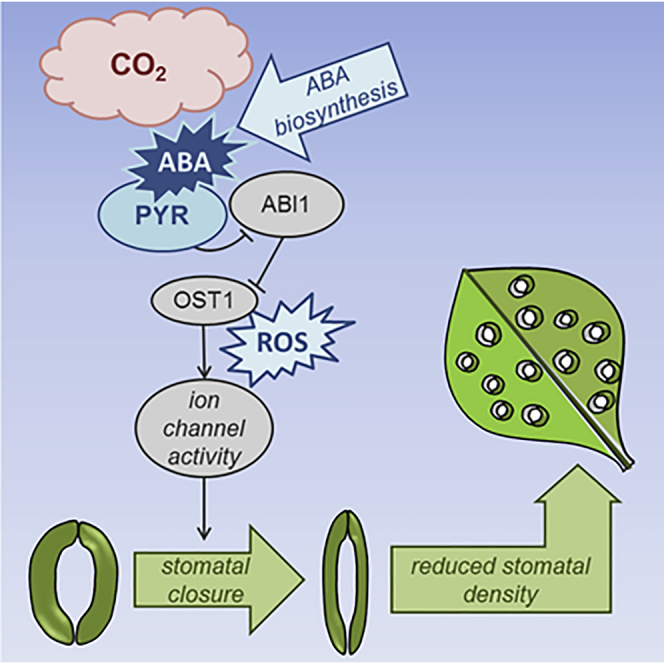

## Teaser

Chater et al. describe the requirement for ABA and ABA signaling in both elevated CO_2_-induced stomatal closure and elevated CO_2_-induced reductions in stomatal density, suggesting that ABA itself is downstream of stomatal CO_2_ perception and that ABA signaling is likely to predate the origin of CO_2_-induced stomatal responses.

## Results and Discussion

The components known to act earliest in the *Arabidopsis* guard cell [CO_2_] signaling pathway that reduce stomatal apertures in response to elevated [CO_2_] are the β-carbonic anhydrases [10]. The HT1 protein kinase is also an early player, the RHC1 MATE transporter plays a role, and in tobacco, a protein kinase NtMPK4 is implicated [11–13], while the Munc13-like protein PATROL1 is involved in low [CO_2_]-induced stomatal opening [14].

Downstream of the HT1 protein kinase there is evidence that the guard cell elevated [CO_2_]-signaling pathway converges with the guard cell ABA-signaling pathway. In 1997, Webb and Hetherington showed, using isolated epidermal preparations, that the *Arabidopsis abi1* and *abi2* mutants, which are defective in guard cell ABA signaling [15], were also compromised in their ability to respond to elevated [CO_2_] [16]. Using similar mutants, Leymarie, Vavasseur, and Lasceve [17] observed that the stomatal opening response to low [CO_2_] was partially disrupted. More recently, using electrophysiological and gas exchange techniques, Merilo et al. [18] concluded that the guard cell response to [CO_2_] is affected in *abi1-1* and *abi2-1*. Several other components of the guard cell ABA signaling pathway have been shown to function in guard cell [CO_2_] signaling [3, 18], including Ca^2+^ [19, 20], the protein kinase OST1, GCA2, and the SLAC1 and ALMT12 anion channels [20–24].

Stomatal development is also controlled by both [CO_2_] and ABA, with stomatal density typically reduced in plants grown under elevated [CO_2_] or following treatment with ABA [25–27]. Although we know much about the basal signaling pathway directing stomatal development [28, 29], we know little about how this pathway is modulated by environmental stimuli [28, 30]. In the case of the reduction in stomatal density that occurs during growth at elevated [CO_2_], in *Arabidopsis* it is known that the putative β-keto acyl CoA synthase HIC plays a role [31] as does the Epidermal Patterning Factor 2 (EPF2) peptide, CO_2_ Response Secreted Protease (CRSP) and β-carbonic anhydrases [10, 32]. Although a role for ABA in the stomatal development response to [CO_2_] has been suggested [33, 34], this has not been directly tested. We decided to investigate whether other known guard cell ABA-signaling components including reactive oxygen species (ROS) [35–37], which increases in response to bicarbonate ions [38], the ABA binding proteins of the PYR/RCAR family [8, 9, 39], and ABA itself are required in either the stomatal aperture or the stomatal density response to elevated [CO_2_].

**Figure 1:**
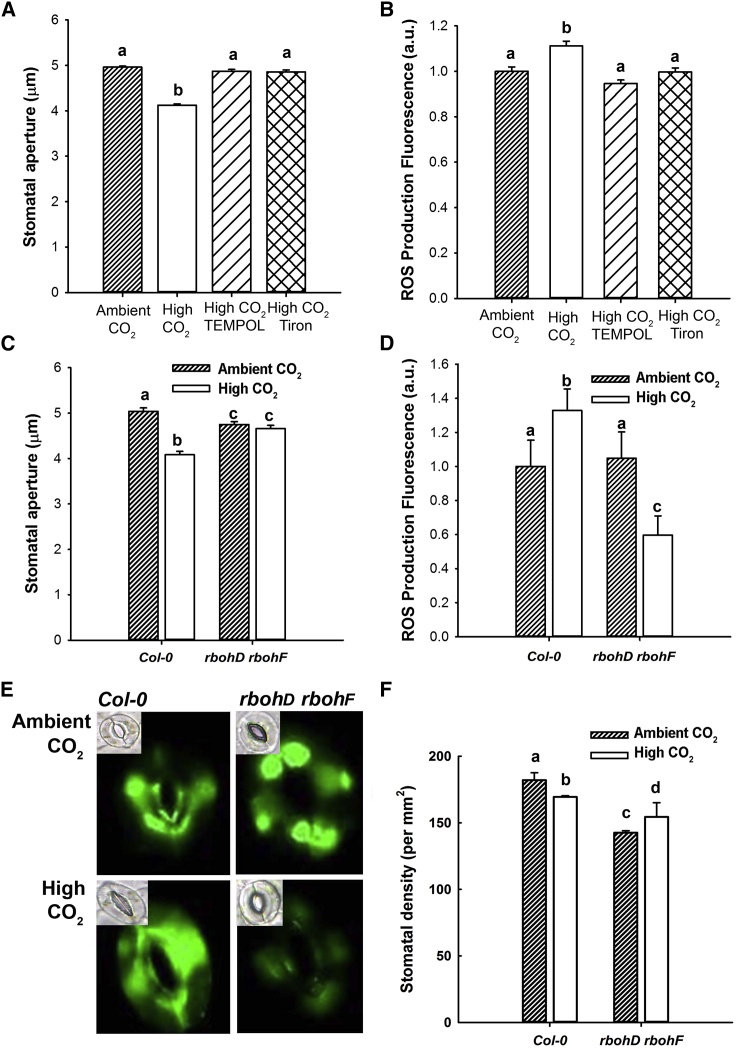
Stomatal Response to Elevated [CO_2_] Requires Generation of Reactive Oxygen Species in Guard Cells (A) Stomatal closure induced by elevated [CO_2_] is inhibited by reactive oxygen species (ROS) scavengers Tiron and Tempol. Mean stomatal aperture is significantly reduced in wild-type stomata treated with 700 ppm [CO_2_] (ANOVA, p < 0.001) compared with treatment with ambient [CO_2_]. This response is disrupted by Tiron or Tempol. Error bars represent SE in this and following figures. (B) Elevated [CO_2_] stimulates an increase in guard cell H_2_DCFDA fluorescence that is blocked in the presence of Tempol or Tiron. Mean fluorescence was significantly higher in stomata treated with 700 ppm [CO_2_] (ANOVA, p < 0.001) compared with treatment with ambient [CO_2_] but did not increase when preparations were pretreated with ROS scavengers. Fluorescence expressed as a.u. relative to wild-type value at ambient [CO_2_]. (C) Elevated [CO_2_]-induced stomatal closure is disrupted in the *rbohD rbohF* mutant. Mean stomatal aperture is significantly reduced in wild-type stomata treated with 1,000 ppm [CO_2_] (ANOVA, p < 0.05) compared with treatment with ambient CO_2_, but not in *rbohD rbohF* stomata. (D) Elevated [CO_2_] stimulates an increase in wild-type guard cell H_2_DCFDA fluorescence but results in decreased fluorescence in *rbohD rbohF* guard cells. Mean fluorescence was significantly higher in wild-type treated with 1,000 ppm [CO_2_] (ANOVA, p < 0.001) compared with treatment with ambient [CO_2_] but decreased in *rbohD rbohF* guard cells. (E) Representative images showing fluorescence of *rbohD rbohF* and wild-type guard cells under ambient and elevated (1,000 ppm) [CO_2_] as in (D). Insets show representative bright-field images used to determine stomatal apertures from (C). (F) The stomatal density response to elevated (1,000 ppm) [CO_2_] requires ROS signaling via NADPH oxidases RbohF and RbohD. Mean stomatal density of wild-type leaves was significantly reduced when grown under 1,000 ppm [CO_2_] in comparison to ambient [CO_2_] (ANOVA, p < 0.001) but was not reduced in *rbohD rbohF* at elevated [CO_2_].

We used the ROS scavengers Tempol [40] and Tiron [41] and the fluorescent ROS indicator H_2_DCFDA [35]. Challenge of guard cells with elevated [CO_2_] resulted in a significant increase in H_2_DCFDA fluorescence, consistent with a [CO_2_]-induced increase in ROS. This increase in fluorescence was blocked in the presence of 10 mM Tempol or Tiron, and the presence of these scavengers also significantly reduced the ability of elevated [CO_2_] to bring about stomatal closure (Figures 1A and 1B), suggesting that an increase in guard cell ROS is required in [CO_2_]-induced stomatal closure.

To confirm that ROS generation is required and to investigate the origin of the ROS, we analyzed [CO_2_]-induced stomatal closure in NADPH respiratory burst oxidase mutants. Previous work has revealed that these enzymes play roles in guard cell ABA signaling, the *rbohD rbohF* double mutant being compromised in guard cell ABA signaling [35]. The results of our experiments (Figure 1C) show that while there was a statistically significant reduction in stomatal aperture elicited by elevated [CO_2_] in wild-type (19% reduction in stomatal aperture), this treatment failed to induce stomatal closure in the *rbohD rbohF* mutant. Although elevated [CO_2_] induced an increase in wild-type guard cell H_2_DCFDA fluorescence (32% increase in fluorescence intensity), in *rbohD rbohF*, no such increase was observed (Figures 1D and 1E). Instead, we observed a strong decrease in H_2_DCFDA fluorescence in *rbohD rbohF* guard cells at elevated [CO_2_], probably explained by a reduction in oxygenase activity of RuBisCO at high [CO_2_], and hence a reduction in H_2_O_2_ production by glycolate oxidase activity linked to photorespiration [42]. To investigate whether ROS signaling is also required for the control of stomatal development by [CO_2_], we grew *rbohD rbohF* plants at ambient and elevated atmospheric [CO_2_]. Wild-type plants developed a significantly lower density of stomata on their mature leaves following growth at elevated [CO_2_] (7% reduction in stomatal density), whereas the *rbohD rbohF* plants exhibited an increased stomatal density (Figure 1F). These results support a role for NADPH oxidase activity and ROS in elevated [CO_2_]-induced reductions in stomatal density and aperture.

**Figure 2:**
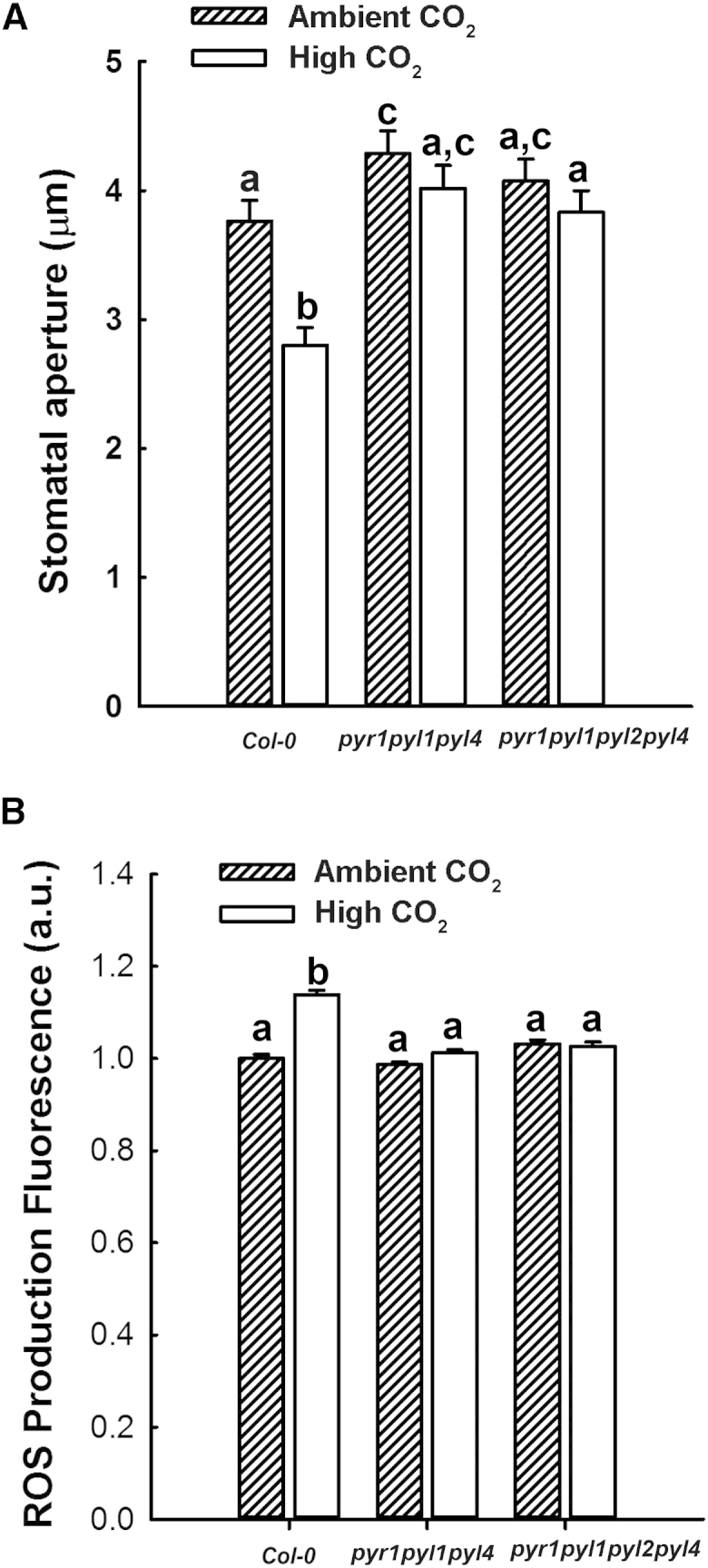
Stomatal Response to Elevated [CO_2_] Requires the PYR/RCAR ABA Receptors (A) Mean stomatal aperture was significantly reduced in wild-type stomata treated with 800 ppm CO_2_ (ANOVA, p < 0.001) compared with treatment with ambient CO_2_, but this response was disrupted in *pyr1 pyl1 pyl4* and *pyr1 pyl1 pyl2 pyl4*. (B) Exposure to elevated [CO_2_] fails to stimulate an increase in guard cell H_2_DCFDA fluorescence in ABA receptor mutants. Mean fluorescence was significantly higher in wild-type stomata treated with 800 ppm [CO_2_] (ANOVA, p < 0.001) compared with treatment with ambient [CO_2_] but did not increase in *pyr1 pyl1 pyl4* and *pyr1 pyl1 pyl2 pyl4*.

Next, we investigated the role of ABA in the guard cell response to elevated [CO_2_]. To do this, we first focused on the possible role of the PYR/RCAR family of ABA receptors [8, 9, 43, 44], specifically PYR1, PYL1, PYL2, and PYL4, which are highly expressed and to some degree functionally redundant in guard cells [39]. The results in Figure 2A show that neither the triple *pyr1 pyl1 pyl4* nor the quadruple *pyr1 pyl1 pyl2 pyl4* [43] ABA receptor mutants exhibited elevated [CO_2_]-mediated reductions in stomatal aperture (under conditions that elicited a 26% reduction in wild-type stomatal aperture). In addition, when we measured elevated [CO_2_]-mediated H_2_DCFDA fluorescence, in contrast to wild-type, which showed a 14% increase in fluorescence, there was no evidence for increased ROS production in either the triple or quadruple ABA receptor mutants (Figure 2B). Taken together, these results suggest that there is a requirement for at least one or more of the *PYR1*, *PYL1*, *PYL2*, and *PYL4* gene products in the [CO_2_]-stimulated increase in ROS that occurs during [CO_2_]-mediated stomatal closure. The involvement of the PYR/RCAR family of ABA receptors in guard cell [CO_2_] signaling has been investigated previously [18]. Xue et al. [21] observed a wild-type response to 800 ppm [CO_2_] in *pyr1 pyl1 pyl2 pyl4*, whereas using gas exchange techniques, Merilo et al. [18] reported a reduced response to high [CO_2_] in the *pyr1 pyl1 pyl2 pyl4 pyl5 pyl8* sextuple mutant. Although the results from Merilo et al. [18] using gas exchange were not as dramatic as the results reported here, some involvement of the ABA receptor family in guard cell [CO_2_] signaling was apparent. Given that Xue et al. [21] reported a wild-type response to high [CO_2_] in the *pyr1 pyl1 pyl2 pyl4* mutant, we repeated this experiment independently in Sheffield and Bristol. The Bristol data (not shown) replicate the Sheffield data shown in Figure 2A. We do not have an explanation for the differences between the Xue et al. [21] data and our own. However, our finding that the elevated [CO_2_]-stimulated increase in ROS (Figure 2B) is absent in the *pyr1 pyl1 pyl4* and *pyr1 pyl1 pyl2 pyl4* guard cells clearly suggests a role for the ABA receptor family in the regulation of NADPH oxidase activity in guard cell [CO_2_] signaling.

**Figure 3:**
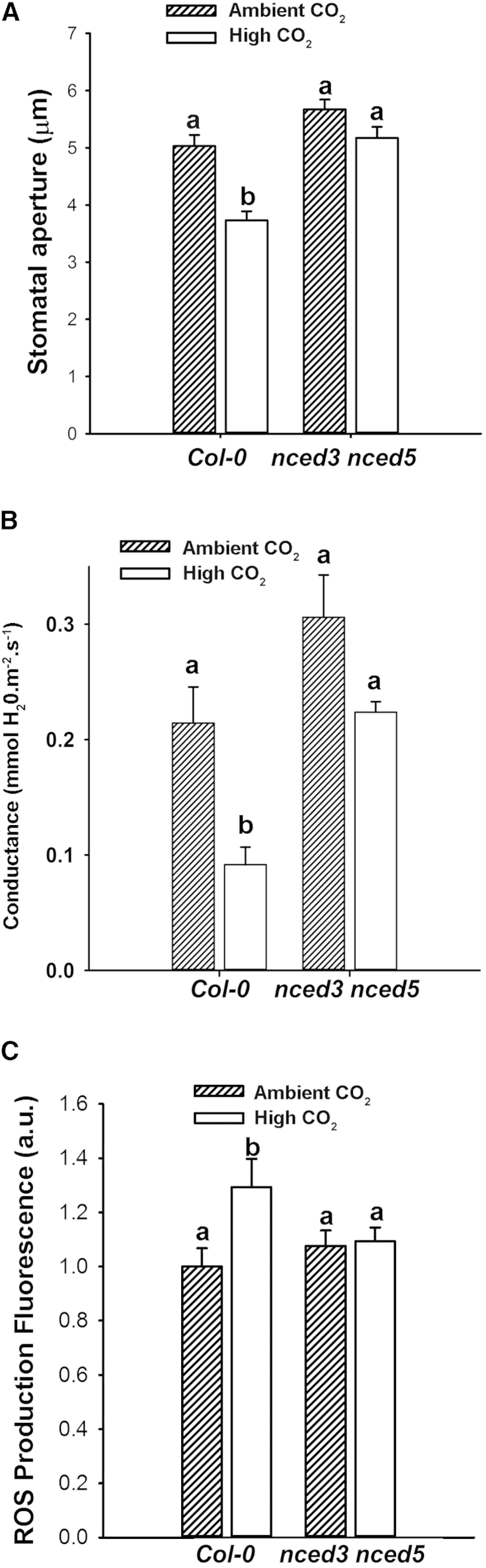
Stomatal Aperture Response to Elevated [CO_2_] Requires ABA Biosynthesis in Guard Cells (A) Mean stomatal aperture is significantly reduced in wild-type stomata treated with 1,000 ppm CO_2_ (ANOVA, p < 0.001), but this response is disrupted in *nced3 nced5*. (B) Exposure to elevated [CO_2_] does not induce a significant reduction in stomatal conductance in *nced3 nced5*. Mean stomatal conductance was significantly reduced in leaves of wild-type plants exposed to 1,000 ppm [CO_2_] (ANOVA, p = 0.0155) compared to ambient [CO_2_], but not in *nced3 nced5* (ANOVA, p = 0.1615). (C) Exposure to elevated [CO_2_] fails to stimulate an increase in guard cell H_2_DCFDA fluorescence in *nced3 nced5*. Mean fluorescence was significantly higher in wild-type stomata treated with 1,000 ppm [CO_2_] (ANOVA, p < 0.01) compared with treatment with ambient [CO_2_] but did not increase in *nced3 nced5*.

The results in Figure 2A show that members of the PYR/RCAR ABA receptor family [45] are required for elevated [CO_2_]-induced stomatal closure; however, they do not shed light on whether ABA itself is involved in this response. Webb and Hetherington [16] took a genetic approach to addressing this question and found that [CO_2_]-mediated stomatal closure in the ABA-deficient mutant *aba1* [46] was similar to wild-type. Similarly, Merilo et al. [18] used *aba1* and *aba3* mutants and showed that [CO_2_]-induced stomatal closure is maintained. These results suggest that [CO_2_]-mediated stomatal closure does not require ABA. However, as leaf ABA levels of *aba1* and *aba3-1* have been reported to be approximately 17% and 10% of corresponding wild-type ABA levels, respectively [46, 47], it is possible that there was sufficient residual ABA in these mutants to satisfy any requirement in [CO_2_] signaling. We re-investigated this issue by assessing elevated [CO_2_]-induced stomatal closure and ROS production in the *nced3 nced5* double mutant, which lacks expression of two guard cell-expressed isoforms of 9-cis-epoxycarotenoid dioxygenase catalyzing the first committal step in ABA biosynthesis [48, 49]. *nced3 nced5* plants are characterized by increased leaf water loss and extremely low ABA levels (approximately 1.5% of wild-type leaf ABA levels [48]). In our experiment (Figure 3A), the stomata of *nced3 nced5* ABA-deficient plants were unable to close significantly in response to elevated [CO_2_] under conditions where wild-type stomatal apertures were reduced by 27%. This striking result suggests either that a CO_2_-induced increase in ABA is required to initiate stomatal closure or that in the presence of reduced ABA, the sensitivity of stomatal closure to elevated [CO_2_] is reduced. We confirmed the result observed in isolated epidermis by using a second technique—infrared gas analysis (Figure 3B), which showed that the ABA-deficient *nced3 nced5* plants were impaired in their ability to adjust their leaf stomatal conductance in response to high [CO_2_]. Furthermore, the experiment shown in Figure 3C reveals that, in contrast to the 29% increase in fluorescence in wild-type stomata, *nced3 nced5* stomata failed to exhibit an increase in elevated [CO_2_]-stimulated H_2_DCFDA fluorescence. The results of these experiments support an essential role for ABA in the response of stomata to increased [CO_2_], either through an absolute requirement for an increase in ABA or through “setting” the sensitivity of the guard cell response to [CO_2_], or indeed both. It will be interesting in future work to investigate the question of synthesis versus sensitivity. Recently developed single cell approaches [50–52,] should make it possible to investigate whether elevated [CO_2_] induces an increase in guard cell ABA (either through de novo synthesis or release from conjugated forms). Similarly, the question of sensitivity could be probed in the *nced3 nced5* background by investigating whether there is a level of exogenously applied ABA that does not promote stomatal closure on its own but that imparts sensitivity of this mutant to [CO_2_].

**Figure 4:**
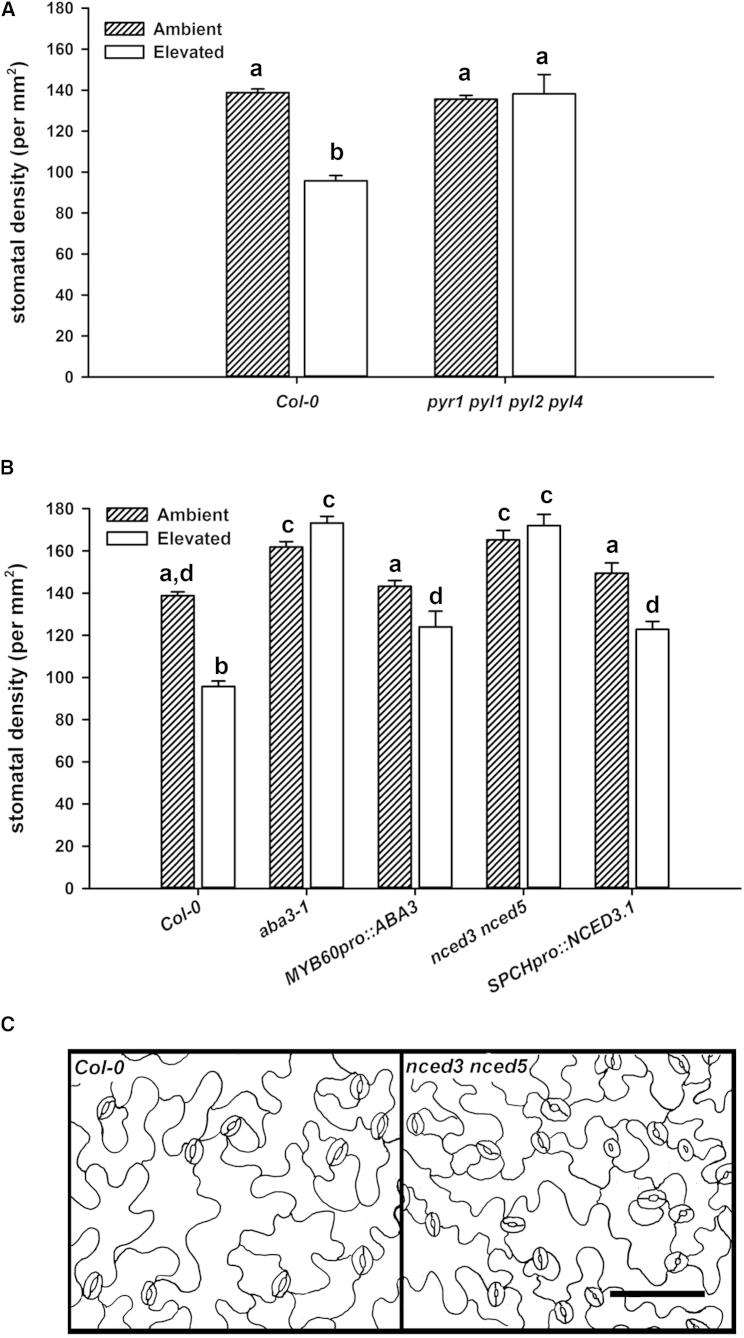
The Stomatal Density Response to [CO_2_] Requires ABA Perception and Biosynthesis (A) Mean stomatal density of wild-type leaves was significantly reduced when grown under 1,000 ppm [CO_2_] in comparison to when grown at ambient [CO_2_] (ANOVA, p < 0.001) but was not reduced in *pyr1 pyl1 pyl2 pyl4* at elevated [CO_2_]. (B) Stomatal densities of *nced3 nced5* and *aba3* were significantly higher than wild-type when grown under either ambient or elevated [CO_2_] (1,000 ppm) (ANOVA, p < 0.001) and did not reduce when grown at elevated [CO_2_]. Stomatal densities of *MYB60*_*pro*_*::ABA3* or *SPCH*_*pro*_*::NCED3-YFP* were not significantly different to wild-type when grown under ambient [CO_2_] but reduced significantly when grown at elevated [CO_2_] (ANOVA, p < 0.05). See also Figure S1. (C) Tracing of epidermal impressions to illustrate the difference in stomatal densities between wild-type and *nced3 nced5* leaves following growth at 1,000 ppm [CO_2_]. The scale bar represents 100 μm.

Next, we showed that ABA perception and presence are also required for regulation of stomatal development by elevated [CO_2_]. We grew plants at ambient and elevated [CO_2_] and found that neither the ABA receptor nor the ABA biosynthesis mutants exhibited elevated [CO_2_]-induced reductions in stomatal density (Figures 4A and 4B), whereas the wild-type plants showed a 32% reduction in stomatal density. Stomatal density in the *nced3 nced5* and *aba3-1* ABA biosynthesis mutants was significantly greater than wild-type at both ambient and elevated [CO_2_], in line with the proposal that ABA is an inhibitor of stomatal development [27, 34]. The differences in stomatal development following growth at elevated [CO_2_] were clear between wild-type and ABA-deficient plants; *nced3 nced5* leaves had 80% increased stomatal density in comparison to wild-type (Figures 4B and 4C). These data suggest that like stomatal aperture, an increase in ABA is required during the reduction in stomatal density induced by exposure to CO_2_ or that the presence of ABA modulates the sensitivity of stomatal development to [CO_2_]. To investigate where and when ABA is required in the elevated [CO_2_]-mediated control of stomatal density, we used ABA-deficient mutants that had rescued ABA biosynthesis in guard cells and stomatal precursor cells. First, we used *MYB60*_*pro*_*::ABA3* plants in which ABA biosynthesis is restored specifically in mature guard cells of the ABA-deficient mutant *aba3-1* (previously demonstrated by using *HVA22* expression as a proxy measure for ABA levels in an investigation of guard cell autonomous ABA production during reduced atmospheric relative humidity-induced stomatal closure [49]). Second, we created *SPCH*_*pro*_*::NCED3-YFP* plants in which ABA biosynthesis is directed to the immature epidermis and stomatal lineage cells [53]. We confirmed elevated levels of *NCED3* expression and ABA in comparison to their *nced3 nced5* background, using qPCR and mass spectrometry (Figures S1A and S1B). As expected, *SPCH*_*pro*_*::NCED3-YFP* expressed NCED3-YFP fluorescent fusion protein in undifferentiated epidermal cells that have the capacity to enter the stomatal lineage and at a lower level in young guard cells (Figures S2A and S2B, respectively). When *MYB60*_*pro*_*::ABA3* or *SPCH*_*pro*_*::NCED3-YFP* plants were grown at ambient [CO_2_], they displayed wild-type stomatal density (Figure 4B) in contrast to their *aba3-1* or *nced3 nced5* backgrounds that both exhibited higher stomatal densities in comparison with wild-type (16% and 19% increases in density, respectively). When either *MYB60*_*pro*_*::ABA3* or *SPCH*_*pro*_*::NCED3-YFP* plants were grown at elevated [CO_2_], there was a reduction in stomatal density compared with the density observed at ambient [CO_2_] (13% and 17% reduction). Although the reductions in stomatal density in response to [CO_2_] were not as great as those observed in the wild-type (31% reduction), they were statistically significant. These data suggest that a reduction in stomatal density at elevated [CO_2_] can be brought about by the specific restoration of ABA synthesis in stomatal precursor cells and/or guard cells. Interestingly, expression of stomatal development regulator *EPF2* [54] was enhanced in the *nced3 nced5* background, perhaps reflecting the increased population of stomatal precursors. This *EPF2* expression was restored to wild-type levels in *SPCH*_*pro*_*::NCED3-YFP* plants under ambient and elevated [CO_2_] (Figure S1). No significant differences were observed in the expression of transcriptional regulators *SPCH* and *MUTE* (Figure S1A). The elevated [CO_2_] responses of two further independently transformed *SPCH*_*pro*_*::NCED3-YFP* lines were tested. Stomatal densities were restored to wild-type levels in all three lines at ambient [CO_2_], and the elevated [CO_2_] response was also restored in two out of three lines (Figure S1D).

Next, we investigated whether an increase in ABA biosynthesis was required for stomatal responses to [CO_2_] but observed no significant difference in ABA levels in the aerial parts of wild-type plants grown under ambient [CO_2_] or elevated [CO_2_] and subsequently subjected to 24-hr reciprocal transfer treatments (Figure S1C). These data are in line with previous observations [55] and consistent with the absence of *NCED3* expression changes in wild-type plants at elevated [CO_2_] (Figure S1A). Of course, these data gathered from bulk aerial tissue do not rule out the possibility that CO_2_ brings about an increase in guard cell ABA levels. Overall, these analyses suggest that it is the sensitivity to or precise localization of the ABA rather than total foliar ABA concentrations per se that are responsible for the stomatal density response to changes in [CO_2_].

In conclusion, we show that both the elevated [CO_2_]-mediated control of stomatal density and aperture require an increase in ROS, thereby adding a new common element to these signaling pathways, and that the elevated [CO_2_]-mediated control of stomatal aperture and stomatal density both require the presence of PYR/RCAR ABA receptors and ABA itself. Our data suggest, in both responses, that [CO_2_]-dependent stomatal responses are conditional on the presence of ABA in that there is an absolute requirement for ABA receptors and ABA. Mechanistically this may be brought about by a [CO_2_]-induced increase in guard cell ABA or a [CO_2_]-induced modulation of the sensitivity of these systems to ABA. This requirement for ABA in [CO_2_]-induced stomatal closure explains why guard cell ABA and [CO_2_] signaling have so many components in common. We suggest that at least some of the effects of [CO_2_] on stomata result from its ability to access the guard cell ABA signaling pathway through the intermediacy of ABA. The point of convergence of ABA and [CO_2_] signaling is controversial [56], and our data point to ABA as being the point of convergence, as might also be true for the stomatal response to relative humidity [49, 57]. It will be interesting to see whether the same is true for other stomatal closure-inducing stimuli.

Finally, in the context of the evolution of stomatal signaling pathways [58–60], our evidence that [CO_2_]-induced stomatal responses require ABA suggests that stomatal ABA responses are in evolutionary terms ancestral to elevated [CO_2_] responses.

## Author Contributions

C.C., M.M., K.P., S.C., H.J.W., and J.A.D. performed experiments and analyzed data. Y.-K.L., D.H.M., J.C.I., I.W., and S.J.N. analyzed and interpreted data. C.C., J.E.G., A.M.H., and R.H. designed experiments, wrote the manuscript, and interpreted data. J.E.G. and A.M.H. conceived the project.

## Supplemental Information

Document S1. Supplemental Experimental Procedures and Figures S1 and S2

Document S2. Article plus Supplemental Information
