# Supplementary material for: Elevated CO2-Induced Responses in Stomata Require ABA and ABA Signaling
Source: Curr Biol. 2015 Oct 19;25(20):2709–16. doi: 10.1016/j.cub.2015.09.013 (PMC4612465; doi:10.1016/j.cub.2015.09.013)
Supplement: Document S1. Supplemental Experimental Procedures and Figures S1 and S2 [file mmc1.pdf]

Current Biology

Supplemental Information

Elevated CO_2-Induced Responses in Stomata Require ABA and ABA Signaling

Caspar Chater, Kai Peng, Mahsa Movahedi, Jessica A. Dunn, Heather J. Walker, Yun-Kuan Liang, Deirdre H. McLachlan, Stuart Casson, Jean Charles Isner, Ian Wilson, Steven J. Neill, Rainer Hedrich, Julie E. Gray, and Alistair M. Hetherington

Supplemental Figure S1, related to Figure 4B.

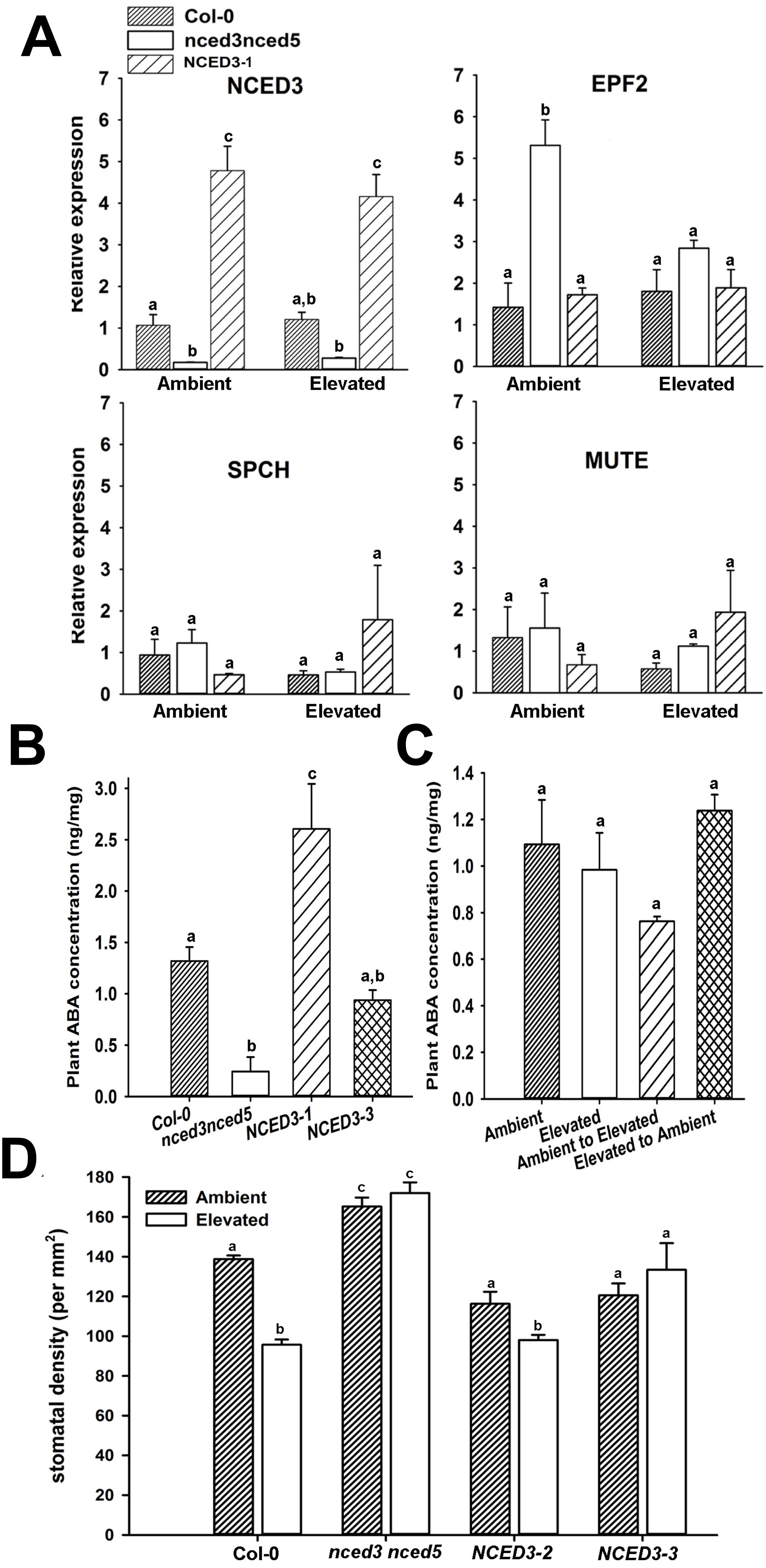

Supplemental Figure S2, related to Figure 4B and Figure S1.

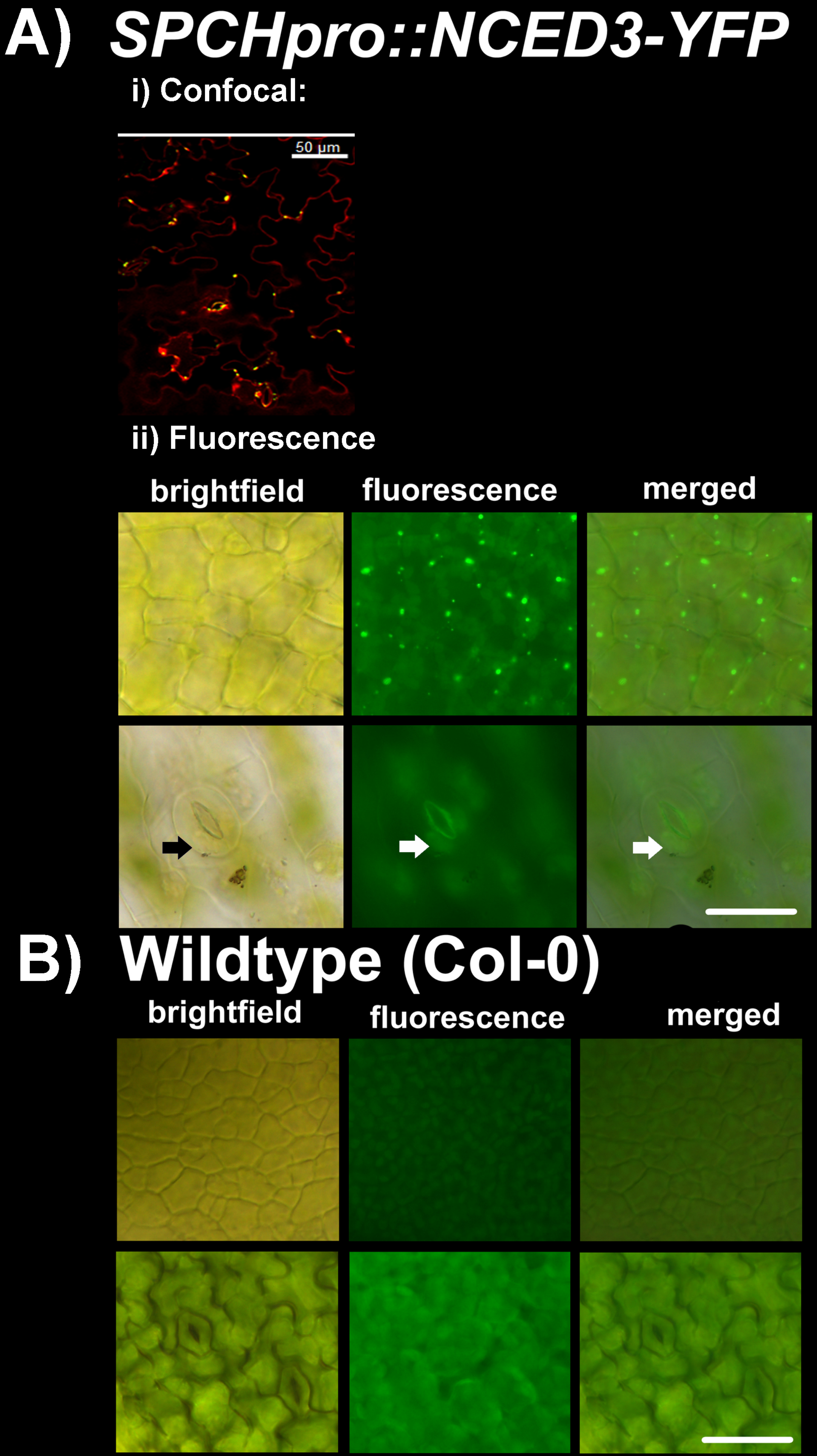

Supplemental Figure Legends

Supplemental Figure S1, related to Figure 4B.

A. qRT-PCR of *NCED3* transcripts showed substantially reduced levels in *nced3 nced5* (ambient[CO_2], ANOVA, p <0.05) which was restored to higher levels in the *SPCHpro::NCED3-YFP* line NCED3-1. Expression of EPF2 was significantly increased in *nced3 nced5* compared with wild-type (ambient [CO_2], ANOVA, p = 0.0016). Expression levels of SPEECHLESS and MUTE were also analysed.
B. ABA levels were significantly reduced in the *nced3 nced5* line compared to wild-type (ANOVA, p = 0.0349) and restored to higher levels in *SPCHpro::NCED3-YFP* lines NCED3-1 and NCED3-3.
C. Levels of ABA measured from wild-type seedlings across [CO_2] growth conditions and reciprocal transfer experiments were not significantly different to each other (ANOVA, p > 0.05).
D. .Stomatal densities of two additional independently transformed lines of *$SPCH_{pro}$::NCED3-YFP*, NCED3-2 and NCED3-3, were not significantly different to wild-type when grown under ambient [CO_2] and density of one (*$SPCH_{pro}$::NCED3*) was significantly reduced when grown at elevated [CO_2] (ANOVA, P <0.001).

Supplemental Figure S2, related to Figure 4B and Figure S1.

A. Fluorescence is detected in the developing epidermis of ten day old *SPCHpro::NCED3-YFP* expressing seedlings in a punctate pattern consistent with localisation to plastids as previously shown by immunolocalization [S1], where the NCED3 step of ABA biosynthesis occurs. (i) Confocal micrograph, with propidium iodide cell wall marker (red), and YFP signal (yellow) (scale = 50 µm), and (ii) brightfield and fluorescence microscopy with overlay (scale = 20 µm). Arrows: a lower level of fluorescence can be observed in chloroplasts of some of the young guard cells in the developing epidermis.

B. YFP fluorescence is not present in wild-type seedling epidermis, shown by brightfield and fluorescence microscopy with overlay (scale = 20 µm).

Supplemental Experimental Procedures

Plant material and growth conditions

Arabidopsis thaliana ecotype Col-0 was used in this investigation. Seeds of *pyr1,pyl1,pyl4* and *pyr1,pyl1,pyl2,pyl4* in the Col-0 background were obtained from Dr Sean Cutler (University of California, Riverside, USA)*, aba3-1*, and *aba3-1::MYB60$_{pro}$::ABA3,* and *nced3 nced5* mutants have been previously described [S2, S3]. For the experiments reported in Figures 1A & B, seeds were surface-sterilised, rinsed, and sown onto a peat based mixed compost of Sinclair multipurpose compost and horticultural silver sand (William Sinclair Horticultural, UK) in a 3:1 ratio in plug-trays. After stratification (4°C in the dark, 2 days), trays were transferred into a Microclima growth cabinet (Snijders Scientific, Netherlands) with a 22°C (day)/ 20°C (night) temperature cycle; 10/14hr light/dark cycle; 70% relative humidity, PPD 120 µmol $m^{-2}s^{-1}$, at ambient [CO_2], approx. 400ppm. 14 day old plants were re-potted into 5 cm Arabaskets in 51-cell plug trays (Beta Tech, Gent, Belgium). For the experiments reported in Figures 1C, D & F, 2, 3, and 4, and Supplemental Figure S1 seeds were sown onto M3 and perlite in a 4:1 ratio in plug-trays and transferred into a growth cabinet (Conviron) with growth conditions 22°C (day)/ 16°C (night) temperature cycle; 9/15hr light/dark cycle; 70% relative humidity, PPD 150 µmol $m^{-2}s^{-1}$, at ambient [CO_2] (approx. 400ppm).

Measurement of stomatal aperture responses

Abaxial epidermis was removed from the youngest, fully expanded leaves of 5 to 6 week old plants and floated, cuticle-side up, on CO_2-free 10 mM MES/KOH (pH 6.15) in 5cm Petri dishes (Sterilin, UK) at 22°C. Epidermal peels were transferred to fresh Petri dishes and incubated for 2h in the light under a PPD of 150 µmol $m^{-2}s^{-1}$ in 50mM KCl, 10 mM MES/KOH (pH 6.15) at 22°C whilst being aerated with CO_2-free air by bubbling through the buffer solution. This treatment brought about stomatal opening. Peels were then either aerated with lab air (ambient, approx. 400 ppm [CO_2]- as measured by IRGA), or elevated [CO_2] from a pressurised cylinder containing CO_2 in air (BOC, Special Gasses, UK) by bubbling directly into the buffer. After 2h peels were removed, mounted on slides and measurements of stomatal aperture recorded using an inverted microscope (Leica DM-IRB, Leica UK) or a microscope (Olympus BX51), fitted camera (Olympus DP70), and ImageJ software v. 1.43u.

Forty stomatal pores were measured per treatment in three separate replicated experiments (total stomatal number = 120; n = 3). To avoid experimenter bias, measurements were performed without the researcher being aware of the sample identity. Additional treatments were as follows: The ROS scavengers Tempol (4-hydroxy-2,2,6,6-tetramethylpiperydine-1-oxyl) (Sigma-Aldrich, UK) and Tiron (4,5-dihydroxy-1,3-benzenedisulfonic acid) (Sigma-Aldrich, UK) were dissolved in water and used at a final concentration of 10 mM [S4] and added immediately prior to the addition of elevated CO_2. Data were analysed using ANOVA (MINITAB and Sigmaplot 12).

ROS and viability assays

To assess viability following the treatments described above, epidermal peels were stained with 0.01 % FDA (Fluorescein Diacetate) (Sigma-Aldrich, UK) from 10 % (w/v) stock solution in ethanol (final ethanol concentration 0.1 % (v/v)) for 10 minutes in dark. Peels were washed in 50mM KCl, 10 mM MES/KOH (pH 6.15) at 22°C for 10 min in the light (PPD 150 µmol $m^{-2}s^{-1}$) and the mean fluorescence intensity (total fluorescence of one guard cell / total area of guard cell) was measured on a Zeiss Axiovert 200M microscope with XBO 75 fluorescent lamp and a GFP filter set fitted with a CCD camera (Hamamatsu) controlled by the Volocity software (Version 5, Improvision). Data were analysed using ANOVA (MINITAB and Sigmaplot 12).

To estimate ROS generation abaxial epidermal peels were prepared as described above and incubated in 50mM KCl, 10 mM MES/KOH (pH 6.15) at 22°C, PPD of 100 µmol $m^{-2}s^{-1}$ whilst being aerated with CO_2-free air for 2h. They were then transferred to 50mM KCl, 10 mM MES/KOH (pH 6.15) at 22°C , PPD of 100 µmol $m^{-2}s^{-1}$ and either aerated with ambient [CO_2] or elevated [CO_2] for 2h. The epidermal peels were then loaded (by pipetting) with 25 µM (final concentration) H_2DCF-DA (2',7'-dichlorodihydrofluorescein diacetate) (Invitrogen, UK) from a 25 mM stock in DMSO for 10 minutes in dark. They were then washed in 50mM KCl, 10 mM MES/KOH (pH 6.15) at 22°C for 10 min in the light (PPD 150 µmol $m^{-2}s^{-1}$) to remove excess H_2DCF-DA and the fluorescence intensity was measured as using an Olympus BX51 fluorescence microscope and ImageJ as described in [S5]. For ROS analysis, pixel intensities of forty stomatal areas (fluorescence zone of two guard cells) relative to their background intensities (four equivalent sized areas surrounding each stoma) were measured per

treatment in three separate replicated experiments (total stomatal number = 120; n = 3). Fluorescence intensities were normalised to those of controls.

Stomatal density measurements

Dental resin (Coltene Whaledent, Switzerland) was applied to the abaxial surfaces of fully expanded leaves and nail varnish peels were taken from set resin. Cell counts were taken from four fields of view from the widest area of four leaves each from at least five plants of each genotype from both growth conditions. Data were analysed using ANOVA (MINITAB and Sigmaplot 12).

Generation of $SPCH_{pro}$*::NCED3* Plants

The Arabidopsis *NCED3* gene was PCR amplified using a 5' primer with overhanging ApaI restriction site (5'- GAGAT TGG GCC CAT GGC TTC TTT CAC GGC A-3') and a 3' primer with overhanging XhoI restriction site (5'- AAA CTC GAG CAC GAC CTG CTT CGC CAA -3'). The *pGKGWY::proSPCH::PHYB* vector [S6] was digested with ApaI and XhoI to remove the *PHYB* gene, and the *NCED3* PCR product was digested with ApaI and XhoI ligated with the *pGKGWY::proSPCH::* backbone to obtain the plasmid *pGKGWY::proSPCH::NCED3* and sequenced. Arabidopsis *nced3 nced5* mutant plants were transformed by vacuum infiltration with *Agrobacterium tumefaciens*, selected for kanamycin resistance. PCR-based confirmation of the stably inserted *proSPCH*::*NCED3-YFP* construct, and RT-PCR and qRT-PCR were carried out to determine *NCED3* expression. F2 generation were used for stomatal analysis.

Gas exchange measurements

Stomatal conductance was measured using infrared gas analysis. Measurements were performed using a portable photosynthesis system attached to a leaf chamber fluorometer with a $2cm^2$ leaf area (LI-COR 6400-40). CO_2 was scrubbed from external air using soda lime and resupplied from a liquid CO_2 cartridge (Liss), to maintain CO_2 concentrations of either 500 or 1000ppm. Relative humidity in the chamber was maintained between 60-75% using self-indicating desiccant (Drierite). Air flow was $200 \mu mol\ s^{-1}$, light intensity was $1000 \mu mol.m^{-2}.s^{-1}$ and the chamber temperature was controlled at 20°C. For each measurement, an individual mature leaf was placed in the leaf chamber, while still attached to the plant.

Photographs of each leaf in the chamber gasket were taken and leaf area used for measuring gas exchange was calculated from these using ImageJ. Leaves were left in the chamber for one hour before measurements were taken, in order to allow them to acclimatise to chamber conditions and for gas exchange to stabilise. Measurements were then logged every 30 seconds for 10 minutes and mean stomatal conductance values taken from these.

Gene Expression

For quantitative RT-PCR analysis (qPCR), RNA was extracted from 2 week old seedlings using the Quick-RNA™ MiniPrep (Zymo Research) plant RNA extraction kit with on-column DNase treatment according to the manufacturer's instructions. RNA was reverse transcribed with High Capacity Reverse Transcriptase (Applied Biosystems). Transcript abundance of target genes was assayed using SYBR Green/JumpStart Taq ReadyMix qPCR Master Mix (Sigma Aldrich). The *ACTIN2* and *UBC21* genes were used as controls, as transcript levels remained constant under all treatments and relative expression levels were calculated using the ΔΔCt method. Expression was calculated relative to that of Col-0 seedlings grown at 400 ppm [CO_2]. Three biological repeats and three technical repeats were performed for each sample and used to calculate s.e.m. values. Reaction conditions were (1 x 95°C - 10 mins; 40 x 95°C - 15s/57°C – 20s/72°C – 30s). Details of primer sequences can be found in supplemental experimental procedures.

**Primer**	**Forward (5'-3')**<br>**Reverse (5'-3')**
SPCH	AACGGTGTCGCATAAGATCC<br>CAAGAGCCAAATCTTCAAGAGC
NCED3	AAAGCCATCGGTGAGCTTCA<br>GCAGCTCTGGCGTAGAATAGC
ACTIN2	TCAGATGCCCAGAAGTGTGTT<br>CCGTACAGATCCTTCCTGATAT
UBC21	GAATGCTTGGAGTCCTGCTTG<br>CTCAGGATGAGCCATCAATGC
EPF2	CCAACATCCTCCCATCCAAGTC<br>TGAGCAATCTGGCAACCTAGACC
MUTE	AACGTCGAAAGACCCTAAACCG<br>TTAGCATGAGGGGAGTTACAGC

ABA quantification by Mass Spectrometry

100 mg of above-ground plant tissue was ground to a powder in liquid nitrogen and homogenised in cold water:chloroform:methanol extraction matrix (80: 200: 470 µl) before vortexing and resting for 30 minutes. 400 µl of cold H_2O was then added before centrifugation at 14000 rpm for 2 minutes. The aqueous phase was transferred to a new tube before an additional 400 µl of cold sterile water was added to re-extract from the chloroform layer. Both aqueous phases were combined, and frozen prior to analysis by mass spectrometry.

ABA concentration was analysed using UPLC-MS Acquity coupled to Synapt G2 mass spectrometer (Waters, UK). Data was collected in negative mode at capillary voltage 2.5 kV, source temperature 120°C, desolvation temperature 350°C, sample cone 20V, extraction cone 4V, desolvation gas flow 900L hr^{-1}, cone gas flow 20L hr^{-1}.UPLC separation was obtained using a linear gradient system (0 minutes 5% acetonitrile in H_20, 3 minutes 35% acetonitrile, 6 minutes 100% acetonitrile, 7.5 minutes 100% acetonitrile, 7.6 minutes 5% acetonitrile) and an Acquity UPLC Peptide BEH C18 column (size= 2.1 mm x 50mm, pore size= 130Å, particle size= 1.7µm) with a flow rate of 0.6 ml min^{-1} and an injection volume of 10µl. Peak size was compared to (+)- ABA standard (Sigma Aldrich).

Confocal microscopy

Confocal images were captured with a Leica TCS SP5 confocal microscope after counterstaining tissues with 10 µg ml^{-1} propidium iodide.

Supplemental References

S1. Endo, A., Sawada, Y., Takahashi, H., Okamoto, M., Ikegami, K., Koiwai, H., Seo, M., Toyomasu, T., Mitsuhashi, W., Shinozaki, K., et al. (2008). Drought Induction of Arabidopsis 9-cis-Epoxycarotenoid Dioxygenase Occurs in Vascular Parenchyma Cells. Plant Phy. *147*, 1984-1993.

S2. Frey, A., Effroy, D., Lefebvre, V., Seo, M., Perreau, F., Berger, A., Sechet, J., To, A., North, H.M., and Marion-Poll, A. (2012). Epoxycarotenoid cleavage by NCED5 fine-tunes ABA accumulation and affects seed dormancy and drought tolerance with other NCED family members. The Plant J. *70*, 501-512.

S3. Bauer, H., Ache, P., Lautner, S., Fromm, J., Hartung, W., Al-Rasheid, Khaled A.S., Sonnewald, S., Sonnewald, U., Kneitz, S., Lachmann, N., et al. (2013). The Stomatal Response to Reduced Relative Humidity Requires Guard Cell-Autonomous ABA Synthesis. Curr. Biol. *23*, 53-57.

S4. Yamada, J., Yoshimura, S., Yamakawa, H., Sawada, M., Nakagawa, M., Hara, S., Kaku, Y., Iwama, T., Naganawa, T., Banno, Y., et al. (2003). Cell permeable ROS scavengers, Tiron and Tempol, rescue PC12 cell death caused by pyrogallol or hypoxia/reoxygenation. Neurosci. Res. *45*, 1-8.

S5. Gavet, O., and Pines, J. (2010). Progressive Activation of CyclinB1-Cdk1 Coordinates Entry to Mitosis. Dev. Cell *18*, 533-543.

S6. Casson, S.A., and Hetherington, A.M. (2014). Phytochrome B Is Required for Light-Mediated Systemic Control of Stomatal Development. Curr. Biol. *24*, 1216-1221.
