# Supplementary material for: Elevated CO2-Induced Responses in Stomata Require ABA and ABA Signaling
Source: Curr Biol. 2015 Oct 19;25(20):2709–16. doi: 10.1016/j.cub.2015.09.013 (PMC4612465; doi:10.1016/j.cub.2015.09.013)
Supplement: Document S2. Article plus Supplemental Information [file mmc2.pdf]

Current Biology

Elevated CO_2-Induced Responses in Stomata Require ABA and ABA Signaling

Graphical Abstract

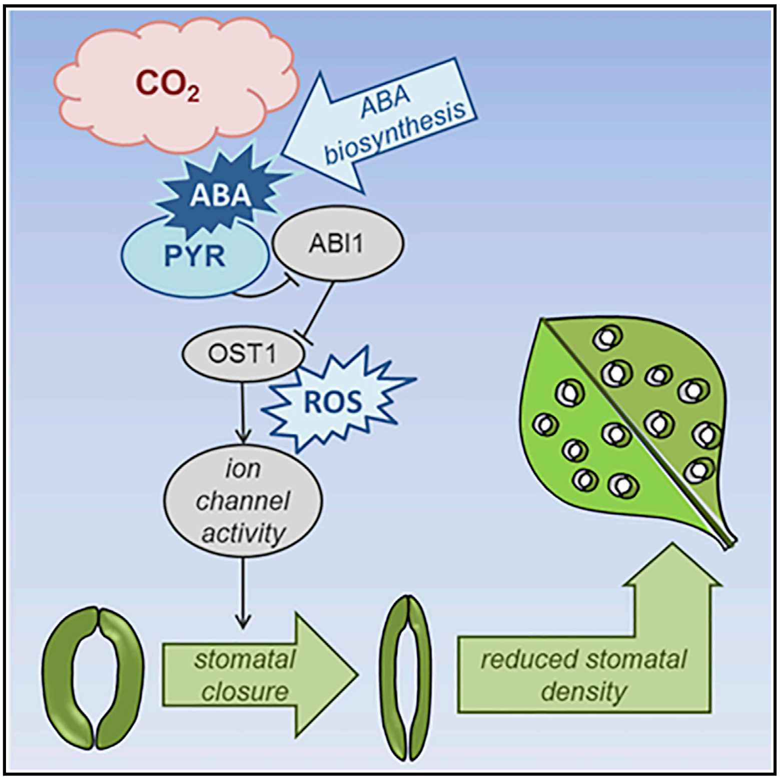

Highlights

- CO_2-induced stomatal closure and density reduction require reactive oxygen species
- CO_2-induced stomatal closure and density reduction require ABA and ABA receptors
- Guard cell/precursor ABA is sufficient to mediate closure and density reduction
- Stomatal CO_2 responses operating via ABA explains overlap between these pathways

Authors

Caspar Chater, Kai Peng, Mahsa Movahedi, ..., Rainer Hedrich, Julie E. Gray, Alistair M. Hetherington

Correspondence

j.e.gray@sheffield.ac.uk (J.E.G.),
alistair.hetherington@bristol.ac.uk (A.M.H.)

In Brief

Chater et al. describe the requirement for ABA and ABA signaling in both elevated CO_2-induced stomatal closure and elevated CO_2-induced reductions in stomatal density, suggesting that ABA itself is downstream of stomatal CO_2 perception and that ABA signaling is likely to predate the origin of CO_2-induced stomatal responses.

Chater et al., 2015, Current Biology *25*, 2709–2716
October 19, 2015 ©2015 The Authors
http://dx.doi.org/10.1016/j.cub.2015.09.013

Current Biology
Report

CellPress

Elevated CO_2-Induced Responses in Stomata Require ABA and ABA Signaling

Caspar Chater,[1] Kai Peng,[2] Mahsa Movahedi,[1] Jessica A. Dunn,[1] Heather J. Walker,[3] Yun-Kuan Liang,[4] Deirdre H. McLachlan,[2,7] Stuart Casson,[1] Jean Charles Isner,[2] Ian Wilson,[5] Steven J. Neill,[5] Rainer Hedrich,[6] Julie E. Gray,[1,*] and Alistair M. Hetherington[2,*]
[1]Department of Molecular Biology and Biotechnology, University of Sheffield, Firth Court, Western Bank, Sheffield S10 2TN, UK
[2]School of Biological Sciences, Life Sciences Building, University of Bristol, Woodland Road, 24 Tyndall Avenue, Bristol BS8 1TQ, UK
[3]Department of Animal and Plant Sciences, University of Sheffield, Alfred Denny Building, Western Bank, Sheffield S10 2TN, UK
[4]State Key Laboratory of Hybrid Rice, College of Life Sciences, Wuhan University, Wuhan 430072, China
[5]Faculty of Health and Life Sciences, University of the West of England, Bristol Frenchay Campus, Coldharbour Lane, Bristol BS16 1QY, UK
[6]Institute for Molecular Plant Physiology and Biophysics, University of Würzburg, 97082 Würzburg, Germany
[7]Present address: The Sainsbury Laboratory, Norwich Research Park, Norwich NR4 7UH, UK
*Correspondence: j.e.gray@sheffield.ac.uk (J.E.G.), alistair.hetherington@bristol.ac.uk (A.M.H.)
http://dx.doi.org/10.1016/j.cub.2015.09.013
This is an open access article under the CC BY license (http://creativecommons.org/licenses/by/4.0/).

SUMMARY

An integral part of global environment change is an increase in the atmospheric concentration of CO_2 ([CO_2]) [1]. Increased [CO_2] reduces leaf stomatal apertures and density of stomata that plays out as reductions in evapotranspiration [2–4]. Surprisingly, given the importance of transpiration to the control of terrestrial water fluxes [5] and plant nutrient acquisition [6], we know comparatively little about the molecular components involved in the intracellular signaling pathways by which [CO_2] controls stomatal development and function [7]. Here, we report that elevated [CO_2]-induced closure and reductions in stomatal density require the generation of reactive oxygen species (ROS), thereby adding a new common element to these signaling pathways. We also show that the PYR/RCAR family of ABA receptors [8, 9] and ABA itself are required in both responses. Using genetic approaches, we show that ABA in guard cells or their precursors is sufficient to mediate the [CO_2]-induced stomatal density response. Taken together, our results suggest that stomatal responses to increased [CO_2] operate through the intermediacy of ABA. In the case of [CO_2]-induced reductions in stomatal aperture, this occurs by accessing the guard cell ABA signaling pathway. In both [CO_2]-mediated responses, our data are consistent with a mechanism in which ABA increases the sensitivity of the system to [CO_2] but could also be explained by requirement for a CO_2-induced increase in ABA biosynthesis specifically in the guard cell lineage. Furthermore, the dependency of stomatal [CO_2] signaling on ABA suggests that the ABA pathway is, in evolutionary terms, likely to be ancestral.

RESULTS AND DISCUSSION

The components known to act earliest in the *Arabidopsis* guard cell [CO_2] signaling pathway that reduce stomatal apertures in response to elevated [CO_2] are the β-carbonic anhydrases [10]. The HT1 protein kinase is also an early player, the RHC1 MATE transporter plays a role, and in tobacco, a protein kinase NtMPK4 is implicated [11–13], while the Munc13-like protein PATROL1 is involved in low [CO_2]-induced stomatal opening [14].

Downstream of the HT1 protein kinase there is evidence that the guard cell elevated [CO_2]-signaling pathway converges with the guard cell ABA-signaling pathway. In 1997, Webb and Hetherington showed, using isolated epidermal preparations, that the *Arabidopsis abi1* and *abi2* mutants, which are defective in guard cell ABA signaling [15], were also compromised in their ability to respond to elevated [CO_2] [16]. Using similar mutants, Leymarie, Vavasseur, and Lasceve [17] observed that the stomatal opening response to low [CO_2] was partially disrupted. More recently, using electrophysiological and gas exchange techniques, Merilo et al. [18] concluded that the guard cell response to [CO_2] is affected in *abi1-1* and *abi2-1*. Several other components of the guard cell ABA signaling pathway have been shown to function in guard cell [CO_2] signaling [3, 18], including Ca^{2+} [19, 20], the protein kinase OST1, GCA2, and the SLAC1 and ALMT12 anion channels [20–24].

Stomatal development is also controlled by both [CO_2] and ABA, with stomatal density typically reduced in plants grown under elevated [CO_2] or following treatment with ABA [25–27]. Although we know much about the basal signaling pathway directing stomatal development [28, 29], we know little about how this pathway is modulated by environmental stimuli [28, 30]. In the case of the reduction in stomatal density that occurs during growth at elevated [CO_2], in *Arabidopsis* it is known that the putative β-keto acyl CoA synthase HIC plays a role [31] as does the Epidermal Patterning Factor 2 (EPF2) peptide, CO_2 Response Secreted Protease (CRSP) and β-carbonic anhydrases [10, 32]. Although a role for ABA in the stomatal development response to [CO_2] has been suggested [33, 34], this has not been directly tested. We decided to investigate whether other

CrossMark

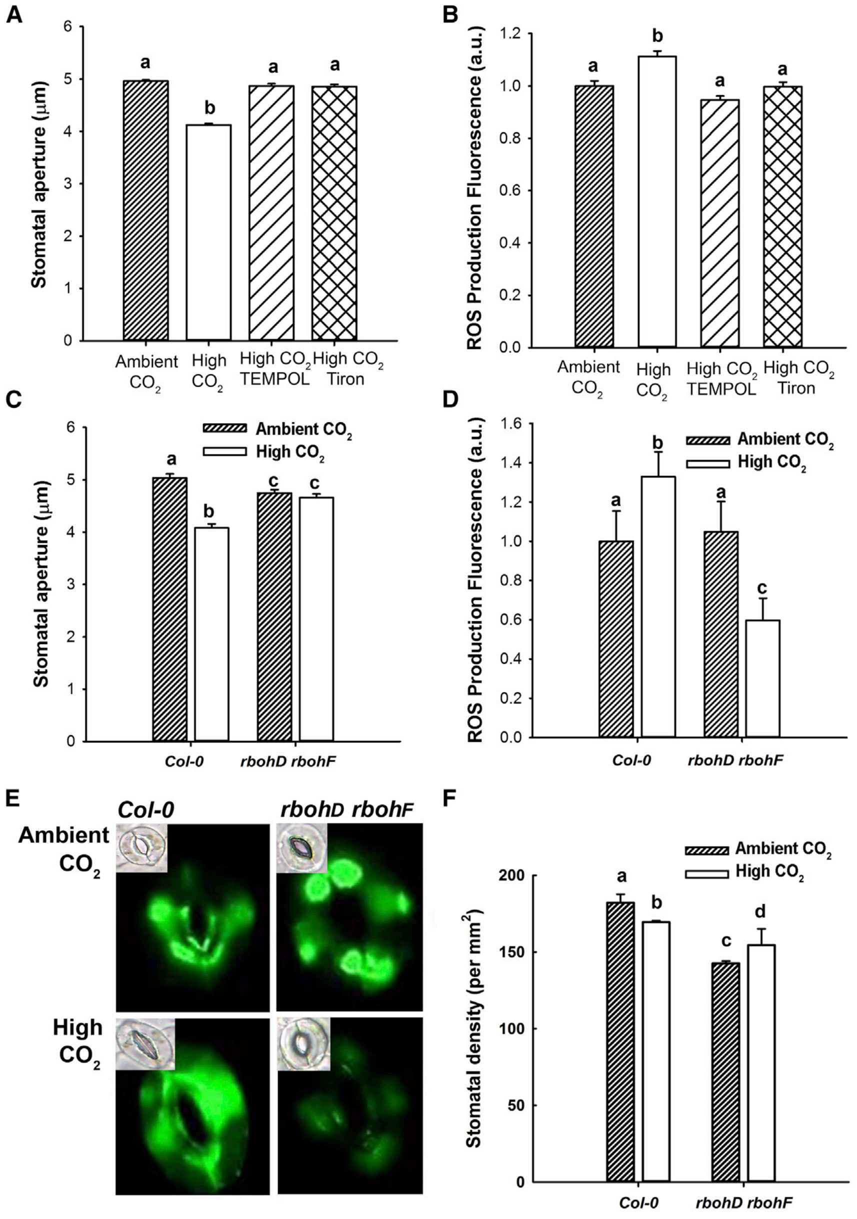

Figure 1. Stomatal Response to Elevated [CO_2] Requires Generation of Reactive Oxygen Species in Guard Cells

(A) Stomatal closure induced by elevated [CO_2] is inhibited by reactive oxygen species (ROS) scavengers Tiron and Tempol. Mean stomatal aperture is significantly reduced in wild-type stomata treated with 700 ppm [CO_2] (ANOVA, $p < 0.001$) compared with treatment with ambient [CO_2]. This response is disrupted by Tiron or Tempol. Error bars represent SE in this and following figures.

(B) Elevated [CO_2] stimulates an increase in guard cell H_2DCFDA fluorescence that is blocked in the presence of Tempol or Tiron. Mean fluorescence was significantly higher in stomata treated with 700 ppm [CO_2] (ANOVA, $p < 0.001$) compared with treatment with ambient [CO_2] but did not increase when preparations were pretreated with ROS scavengers. Fluorescence expressed as a.u. relative to wild-type value at ambient [CO_2].

(C) Elevated [CO_2]-induced stomatal closure is disrupted in the *rbohD rbohF* mutant. Mean stomatal aperture is significantly reduced in wild-type stomata treated with 1,000 ppm [CO_2] (ANOVA, $p < 0.05$) compared with treatment with ambient CO_2, but not in *rbohD rbohF* stomata.

(D) Elevated [CO_2] stimulates an increase in wild-type guard cell H_2DCFDA fluorescence but results in decreased fluorescence in *rbohD rbohF* guard cells. Mean fluorescence was significantly higher in wild-type treated with 1,000 ppm [CO_2] (ANOVA, $p < 0.001$) compared with treatment with ambient [CO_2] but decreased in *rbohD rbohF* guard cells.

(E) Representative images showing fluorescence of *rbohD rbohF* and wild-type guard cells under ambient and elevated (1,000 ppm) [CO_2] as in (D). Insets show representative bright-field images used to determine stomatal apertures from (C).

(F) The stomatal density response to elevated (1,000 ppm) [CO_2] requires ROS signaling via NADPH oxidases RbohF and RbohD. Mean stomatal density of wild-type leaves was significantly reduced when grown under 1,000 ppm [CO_2] in comparison to ambient [CO_2] (ANOVA, $p < 0.001$) but was not reduced in *rbohD rbohF* at elevated [CO_2].

known guard cell ABA-signaling components including reactive oxygen species (ROS) [35–37], which increases in response to bicarbonate ions [38], the ABA binding proteins of the PYR/RCAR family [8, 9, 39], and ABA itself are required in either the stomatal aperture or the stomatal density response to elevated [CO_2].

We used the ROS scavengers Tempol [40] and Tiron [41] and the fluorescent ROS indicator H_2DCFDA [35]. Challenge of guard cells with elevated [CO_2] resulted in a significant increase in H_2DCFDA fluorescence, consistent with a [CO_2]-induced increase in ROS. This increase in fluorescence was blocked in the presence of 10 mM Tempol or Tiron, and the presence of these scavengers also significantly reduced the ability of elevated [CO_2] to bring about stomatal closure (Figures 1A and 1B), suggesting that an increase in guard cell ROS is required in [CO_2]-induced stomatal closure.

To confirm that ROS generation is required and to investigate the origin of the ROS, we analyzed [CO_2]-induced stomatal closure in NADPH respiratory burst oxidase mutants. Previous work has revealed that these enzymes play roles in guard cell ABA signaling, the *rbohD rbohF* double mutant being compromised in guard cell ABA signaling [35]. The results of our experiments (Figure 1C) show that while there was a statistically significant reduction in stomatal aperture elicited by elevated [CO_2] in wild-type (19% reduction in stomatal aperture), this treatment failed to induce stomatal closure in the *rbohD rbohF* mutant. Although elevated [CO_2] induced an increase in wild-type guard cell H_2DCFDA fluorescence (32% increase in fluorescence intensity), in *rbohD rbohF*, no such increase was observed (Figures 1D and 1E). Instead, we observed a strong decrease in H_2DCFDA fluorescence in *rbohD rbohF* guard cells at elevated [CO_2],

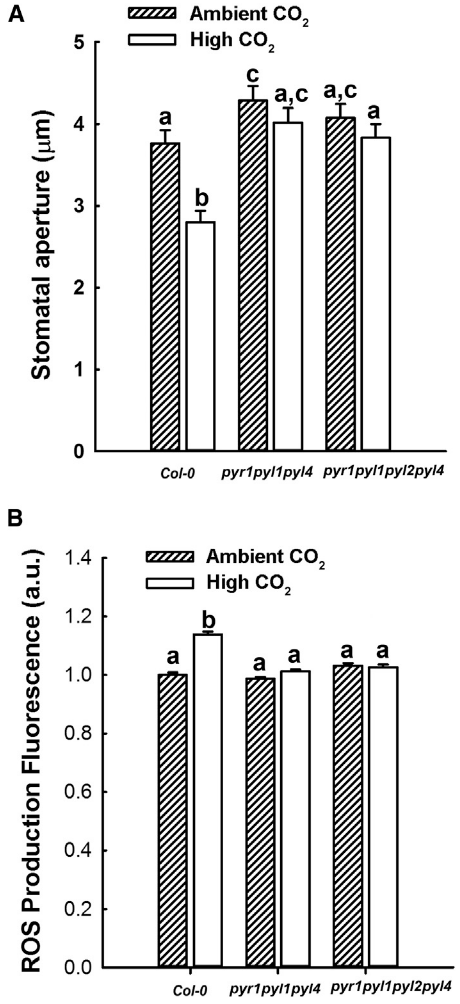

Figure 2. Stomatal Response to Elevated [CO_2] Requires the PYR/RCAR ABA Receptors

(A) Mean stomatal aperture was significantly reduced in wild-type stomata treated with 800 ppm CO_2 (ANOVA, $p < 0.001$) compared with treatment with ambient CO_2, but this response was disrupted in *pyr1 pyl1 pyl4* and *pyr1 pyl1 pyl2 pyl4*.

(B) Exposure to elevated [CO_2] fails to stimulate an increase in guard cell H_2DCFDA fluorescence in ABA receptor mutants. Mean fluorescence was significantly higher in wild-type stomata treated with 800 ppm [CO_2] (ANOVA, $p < 0.001$) compared with treatment with ambient [CO_2] but did not increase in *pyr1 pyl1 pyl4* and *pyr1 pyl1 pyl2 pyl4*.

probably explained by a reduction in oxygenase activity of RuBisCO at high [CO_2], and hence a reduction in H_2O_2 production by glycolate oxidase activity linked to photorespiration [42]. To investigate whether ROS signaling is also required for the control of stomatal development by [CO_2], we grew *rbohD rbohF* plants at ambient and elevated atmospheric [CO_2]. Wild-type plants developed a significantly lower density of stomata on their mature leaves following growth at elevated [CO_2] (7% reduction in stomatal density), whereas the *rbohD rbohF* plants exhibited an increased stomatal density (Figure 1F). These results support a role for NADPH oxidase activity and ROS in elevated [CO_2]-induced reductions in stomatal density and aperture.

Next, we investigated the role of ABA in the guard cell response to elevated [CO_2]. To do this, we first focused on the possible role of the PYR/RCAR family of ABA receptors [8, 9, 43, 44], specifically PYR1, PYL1, PYL2, and PYL4, which are highly expressed and to some degree functionally redundant in guard cells [39]. The results in Figure 2A show that neither the triple *pyr1 pyl1 pyl4* nor the quadruple *pyr1 pyl1 pyl2 pyl4* [43] ABA receptor mutants exhibited elevated [CO_2]-mediated reductions in stomatal aperture (under conditions that elicited a 26% reduction in wild-type stomatal aperture). In addition, when we measured elevated [CO_2]-mediated H_2DCFDA fluorescence, in contrast to wild-type, which showed a 14% increase in fluorescence, there was no evidence for increased ROS production in either the triple or quadruple ABA receptor mutants (Figure 2B). Taken together, these results suggest that there is a requirement for at least one or more of the *PYR1*, *PYL1*, *PYL2*, and *PYL4* gene products in the [CO_2]-stimulated increase in ROS that occurs during [CO_2]-mediated stomatal closure. The involvement of the PYR/RCAR family of ABA receptors in guard cell [CO_2] signaling has been investigated previously [18]. Xue et al. [21] observed a wild-type response to 800 ppm [CO_2] in *pyr1 pyl1 pyl2 pyl4*, whereas using gas exchange techniques, Merilo et al. [18] reported a reduced response to high [CO_2] in the *pyr1 pyl1 pyl2 pyl4 pyl5 pyl8* sextuple mutant. Although the results from Merilo et al. [18] using gas exchange were not as dramatic as the results reported here, some involvement of the ABA receptor family in guard cell [CO_2] signaling was apparent. Given that Xue et al. [21] reported a wild-type response to high [CO_2] in the *pyr1 pyl1 pyl2 pyl4* mutant, we repeated this experiment independently in Sheffield and Bristol. The Bristol data (not shown) replicate the Sheffield data shown in Figure 2A. We do not have an explanation for the differences between the Xue et al. [21] data and our own. However, our finding that the elevated [CO_2]-stimulated increase in ROS (Figure 2B) is absent in the *pyr1 pyl1 pyl4* and *pyr1 pyl1 pyl2 pyl4* guard cells clearly suggests a role for the ABA receptor family in the regulation of NADPH oxidase activity in guard cell [CO_2] signaling.

The results in Figure 2A show that members of the PYR/RCAR ABA receptor family [45] are required for elevated [CO_2]-induced stomatal closure; however, they do not shed light on whether ABA itself is involved in this response. Webb and Hetherington [16] took a genetic approach to addressing this question and found that [CO_2]-mediated stomatal closure in the ABA-deficient mutant *aba1* [46] was similar to wild-type. Similarly, Merilo et al. [18] used *aba1* and *aba3* mutants and showed that [CO_2]-induced stomatal closure is maintained. These results suggest that [CO_2]-mediated stomatal closure does not require ABA. However, as leaf ABA levels of *aba1* and *aba3-1* have been reported to be approximately 17% and 10% of corresponding wild-type ABA levels, respectively [46, 47], it is possible that there was sufficient residual ABA in these mutants to satisfy any requirement in [CO_2] signaling. We re-investigated this issue by assessing elevated [CO_2]-induced stomatal closure and ROS production in the *nced3 nced5* double mutant, which lacks expression of two guard cell-expressed isoforms of 9-cis-epoxycarotenoid dioxygenase catalyzing the first committal step in ABA biosynthesis [48, 49]. *nced3 nced5* plants are characterized by increased leaf water loss and extremely low ABA levels (approximately 1.5% of wild-type leaf ABA levels [48]). In our

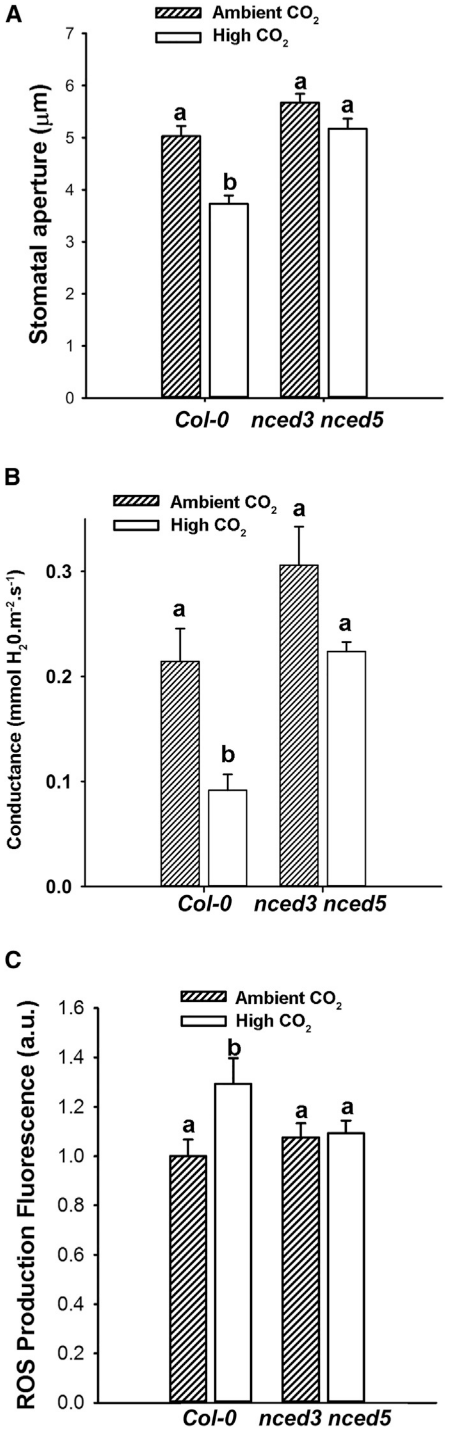

Figure 3. Stomatal Aperture Response to Elevated [CO_2] Requires ABA Biosynthesis in Guard Cells

(A) Mean stomatal aperture is significantly reduced in wild-type stomata treated with 1,000 ppm CO_2 (ANOVA, $p < 0.001$), but this response is disrupted in *nced3 nced5*.

(B) Exposure to elevated [CO_2] does not induce a significant reduction in stomatal conductance in *nced3 nced5*. Mean stomatal conductance was significantly reduced in leaves of wild-type plants exposed to 1,000 ppm [CO_2] (ANOVA, $p = 0.0155$) compared to ambient [CO_2], but not in *nced3 nced5* (ANOVA, $p = 0.1615$).

(C) Exposure to elevated [CO_2] fails to stimulate an increase in guard cell H_2DCFDA fluorescence in *nced3 nced5*. Mean fluorescence was significantly higher in wild-type stomata treated with 1,000 ppm [CO_2] (ANOVA, $p < 0.01$) compared with treatment with ambient [CO_2] but did not increase in *nced3 nced5*.

experiment (Figure 3A), the stomata of *nced3 nced5* ABA-deficient plants were unable to close significantly in response to elevated [CO_2] under conditions where wild-type stomatal apertures were reduced by 27%. This striking result suggests either that a CO_2-induced increase in ABA is required to initiate stomatal closure or that in the presence of reduced ABA, the sensitivity of stomatal closure to elevated [CO_2] is reduced. We confirmed the result observed in isolated epidermis by using a second technique—infrared gas analysis (Figure 3B), which showed that the ABA-deficient *nced3 nced5* plants were impaired in their ability to adjust their leaf stomatal conductance in response to high [CO_2]. Furthermore, the experiment shown in Figure 3C reveals that, in contrast to the 29% increase in fluorescence in wild-type stomata, *nced3 nced5* stomata failed to exhibit an increase in elevated [CO_2]-stimulated H_2DCFDA fluorescence. The results of these experiments support an essential role for ABA in the response of stomata to increased [CO_2], either through an absolute requirement for an increase in ABA or through "setting" the sensitivity of the guard cell response to [CO_2], or indeed both. It will be interesting in future work to investigate the question of synthesis versus sensitivity. Recently developed single cell approaches [50–52,] should make it possible to investigate whether elevated [CO_2] induces an increase in guard cell ABA (either through de novo synthesis or release from conjugated forms). Similarly, the question of sensitivity could be probed in the *nced3 nced5* background by investigating whether there is a level of exogenously applied ABA that does not promote stomatal closure on its own but that imparts sensitivity of this mutant to [CO_2].

Next, we showed that ABA perception and presence are also required for regulation of stomatal development by elevated [CO_2]. We grew plants at ambient and elevated [CO_2] and found that neither the ABA receptor nor the ABA biosynthesis mutants exhibited elevated [CO_2]-induced reductions in stomatal density (Figures 4A and 4B), whereas the wild-type plants showed a 32% reduction in stomatal density. Stomatal density in the *nced3 nced5* and *aba3-1* ABA biosynthesis mutants was significantly greater than wild-type at both ambient and elevated [CO_2], in line with the proposal that ABA is an inhibitor of stomatal development [27, 34]. The differences in stomatal development following growth at elevated [CO_2] were clear between wild-type and ABA-deficient plants; *nced3 nced5* leaves had 80% increased stomatal density in comparison to wild-type (Figures 4B and 4C). These data suggest that like stomatal aperture, an increase in ABA is required during the reduction in stomatal density induced by exposure to CO_2 or that the presence of ABA modulates the sensitivity of stomatal development to [CO_2]. To investigate where and when ABA is required in the elevated [CO_2]-mediated control of stomatal density, we used ABA-deficient mutants that had rescued ABA biosynthesis in guard cells and stomatal precursor cells. First, we used $MYB60_{pro}$::*ABA3* plants in which ABA biosynthesis is restored specifically in mature guard cells of the ABA-deficient mutant *aba3-1* (previously demonstrated

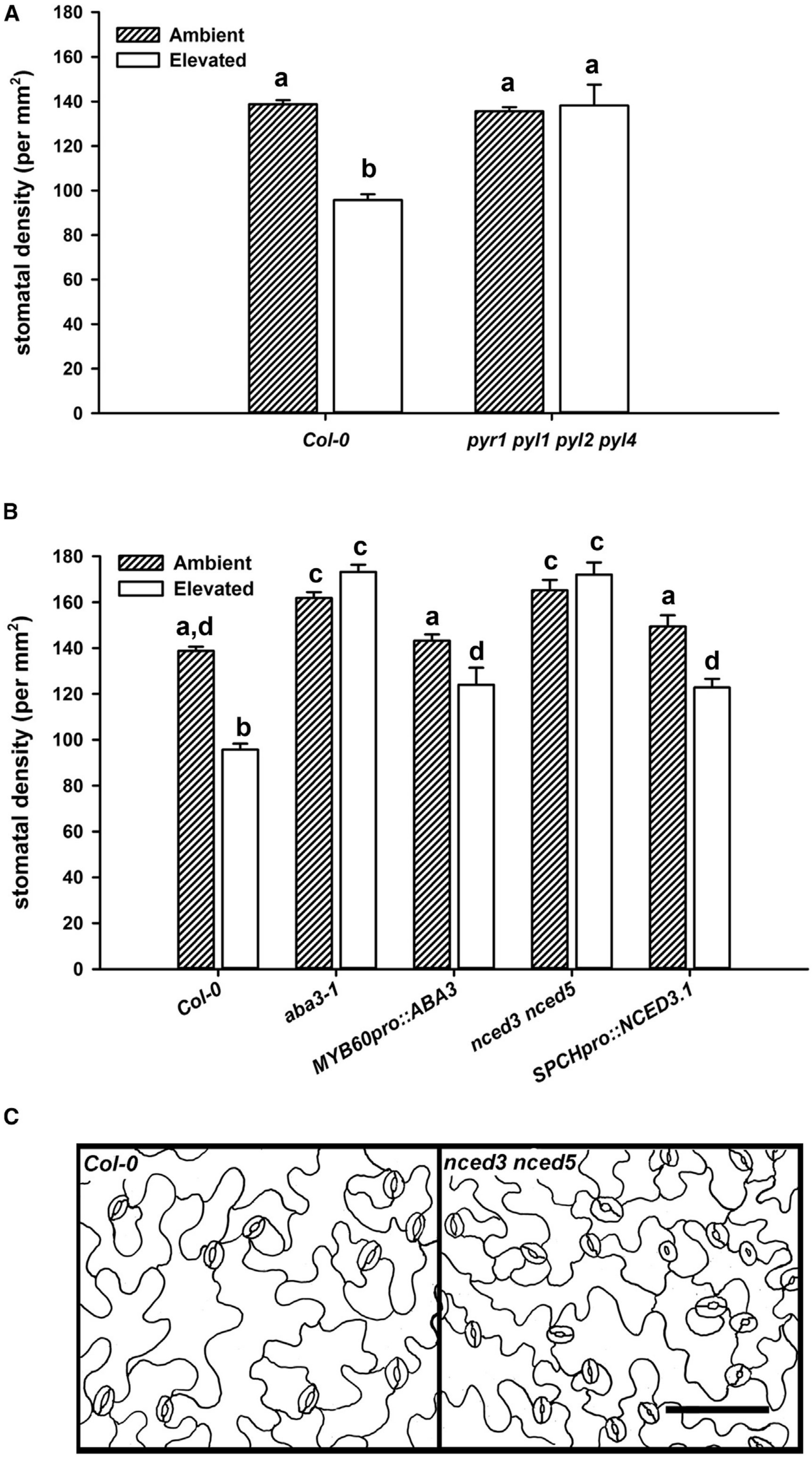

Figure 4. The Stomatal Density Response to $[CO_2]$ Requires ABA Perception and Biosynthesis

(A) Mean stomatal density of wild-type leaves was significantly reduced when grown under 1,000 ppm $[CO_2]$ in comparison to when grown at ambient $[CO_2]$ (ANOVA, $p < 0.001$) but was not reduced in *pyr1 pyl1 pyl2 pyl4* at elevated $[CO_2]$.

(B) Stomatal densities of *nced3 nced5* and *aba3* were significantly higher than wild-type when grown under either ambient or elevated $[CO_2]$ (1,000 ppm) (ANOVA, $p < 0.001$) and did not reduce when grown at elevated $[CO_2]$. Stomatal densities of *MYB60$_{pro}$::ABA3* or *SPCH$_{pro}$::NCED3-YFP* were not significantly different to wild-type when grown under ambient $[CO_2]$ but reduced significantly when grown at elevated $[CO_2]$ (ANOVA, $p < 0.05$). See also Figure S1.

(C) Tracing of epidermal impressions to illustrate the difference in stomatal densities between wild-type and *nced3 nced5* leaves following growth at 1,000 ppm $[CO_2]$. The scale bar represents 100 μm.

by using *HVA22* expression as a proxy measure for ABA levels in an investigation of guard cell autonomous ABA production during reduced atmospheric relative humidity-induced stomatal closure [49]). Second, we created *SPCH$_{pro}$::NCED3-YFP* plants in which ABA biosynthesis is directed to the immature epidermis and stomatal lineage cells [53]. We confirmed elevated levels of *NCED3* expression and ABA in comparison to their *nced3 nced5* background, using qPCR and mass spectrometry (Figures S1A and S1B). As expected, *SPCH$_{pro}$::NCED3-YFP* expressed NCED3-YFP fluorescent fusion protein in undifferentiated epidermal cells that have the capacity to enter the stomatal lineage and at a lower level in young guard cells (Figures S2A and S2B, respectively). When *MYB60$_{pro}$::ABA3* or *SPCH$_{pro}$::NCED3-YFP* plants were grown at ambient $[CO_2]$, they displayed wild-type stomatal density (Figure 4B) in contrast to their *aba3-1* or *nced3 nced5* backgrounds that both exhibited higher stomatal densities in comparison with wild-type (16% and 19% increases in density, respectively). When either *MYB60$_{pro}$::ABA3* or *SPCH$_{pro}$::NCED3-YFP* plants were grown at elevated $[CO_2]$, there was a reduction in stomatal density compared with the density observed at ambient $[CO_2]$ (13% and 17% reduction). Although the reductions in stomatal density in response to $[CO_2]$ were not as great as those observed in the wild-type (31% reduction), they were statistically significant. These data suggest that a reduction in stomatal density at elevated $[CO_2]$ can be brought about by the specific restoration of ABA synthesis in stomatal precursor cells and/or guard cells. Interestingly, expression of stomatal development regulator *EPF2* [54] was enhanced in the *nced3 nced5* background,

perhaps reflecting the increased population of stomatal precursors. This *EPF2* expression was restored to wild-type levels in *SPCH*$_{pro}$*::NCED3-YFP* plants under ambient and elevated [CO_2] (Figure S1). No significant differences were observed in the expression of transcriptional regulators *SPCH* and *MUTE* (Figure S1A). The elevated [CO_2] responses of two further independently transformed *SPCH*$_{pro}$*::NCED3-YFP* lines were tested. Stomatal densities were restored to wild-type levels in all three lines at ambient [CO_2], and the elevated [CO_2] response was also restored in two out of three lines (Figure S1D).

Next, we investigated whether an increase in ABA biosynthesis was required for stomatal responses to [CO_2] but observed no significant difference in ABA levels in the aerial parts of wild-type plants grown under ambient [CO_2] or elevated [CO_2] and subsequently subjected to 24-hr reciprocal transfer treatments (Figure S1C). These data are in line with previous observations [55] and consistent with the absence of *NCED3* expression changes in wild-type plants at elevated [CO_2] (Figure S1A). Of course, these data gathered from bulk aerial tissue do not rule out the possibility that CO_2 brings about an increase in guard cell ABA levels. Overall, these analyses suggest that it is the sensitivity to or precise localization of the ABA rather than total foliar ABA concentrations per se that are responsible for the stomatal density response to changes in [CO_2].

In conclusion, we show that both the elevated [CO_2]-mediated control of stomatal density and aperture require an increase in ROS, thereby adding a new common element to these signaling pathways, and that the elevated [CO_2]-mediated control of stomatal aperture and stomatal density both require the presence of PYR/RCAR ABA receptors and ABA itself. Our data suggest, in both responses, that [CO_2]-dependent stomatal responses are conditional on the presence of ABA in that there is an absolute requirement for ABA receptors and ABA. Mechanistically this may be brought about by a [CO_2]-induced increase in guard cell ABA or a [CO_2]-induced modulation of the sensitivity of these systems to ABA. This requirement for ABA in [CO_2]-induced stomatal closure explains why guard cell ABA and [CO_2] signaling have so many components in common. We suggest that at least some of the effects of [CO_2] on stomata result from its ability to access the guard cell ABA signaling pathway through the intermediacy of ABA. The point of convergence of ABA and [CO_2] signaling is controversial [56], and our data point to ABA as being the point of convergence, as might also be true for the stomatal response to relative humidity [49, 57]. It will be interesting to see whether the same is true for other stomatal closure-inducing stimuli.

Finally, in the context of the evolution of stomatal signaling pathways [58–60], our evidence that [CO_2]-induced stomatal responses require ABA suggests that stomatal ABA responses are in evolutionary terms ancestral to elevated [CO_2] responses.

SUPPLEMENTAL INFORMATION

Supplemental Information includes Supplemental Experimental Procedures and two figures and can be found with this article online at http://dx.doi.org/10.1016/j.cub.2015.09.013.

AUTHOR CONTRIBUTIONS

C.C., M.M., K.P., S.C., H.J.W., and J.A.D. performed experiments and analyzed data. Y.-K.L., D.H.M., J.C.I., I.W., and S.J.N. analyzed and interpreted data. C.C., J.E.G., A.M.H., and R.H. designed experiments, wrote the manuscript, and interpreted data. J.E.G. and A.M.H. conceived the project.

ACKNOWLEDGMENTS

The authors are grateful to Dr. Sean Cutler (University of California) for the gift of the ABA receptor mutants and Dr. Annie Marion-Poll (INRA) for the gift of the ABA biosynthesis mutants. A.M.H. and J.E.G. acknowledge the Biotechnology and Biological Sciences Research Council (BBSRC) and the World Universities Network for supporting the work described in this paper. A.M.H. is grateful to the Royal Society and Leverhulme Trust for the award of a Senior Research Fellowship and the Gatsby Charitable Trust for assistance with buying equipment. Y.-K.L. is also grateful for the financial support from National Natural Science Foundation of China (#31171356). The authors also acknowledge the suggestions of an anonymous reviewer concerning future experiments.

Received: February 16, 2015
Revised: July 21, 2015
Accepted: September 2, 2015
Published: October 8, 2015

REFERENCES

1. Long, S.P., Ainsworth, E.A., Rogers, A., and Ort, D.R. (2004). Rising atmospheric carbon dioxide: plants FACE the future. Annu. Rev. Plant Biol. *55*, 591–628.
2. Mansfield, T.A., Hetherington, A.M., and Atkinson, C.J. (1990). Some current aspects of stomatal physiology. Annu. Rev. Plant Physiol. Plant Mol. Biol. *41*, 55–75.
3. Kim, T.-H., Böhmer, M., Hu, H., Nishimura, N., and Schroeder, J.I. (2010). Guard cell signal transduction network: advances in understanding abscisic acid, CO_2, and Ca^{2+} signaling. Annu. Rev. Plant Biol. *61*, 561–591.
4. Vavasseur, A., and Raghavendra, A.S. (2005). Guard cell metabolism and CO_2 sensing. New Phytol. *165*, 665–682.
5. Jasechko, S., Sharp, Z.D., Gibson, J.J., Birks, S.J., Yi, Y., and Fawcett, P.J. (2013). Terrestrial water fluxes dominated by transpiration. Nature *496*, 347–350.
6. McGrath, J.M., and Lobell, D.B. (2013). Reduction of transpiration and altered nutrient allocation contribute to nutrient decline of crops grown in elevated CO($_2$) concentrations. Plant Cell Environ. *36*, 697–705.
7. Negi, J., Hashimoto-Sugimoto, M., Kusumi, K., and Iba, K. (2014). New approaches to the biology of stomatal guard cells. Plant Cell Physiol. *55*, 241–250.
8. Ma, Y., Szostkiewicz, I., Korte, A., Moes, D., Yang, Y., Christmann, A., and Grill, E. (2009). Regulators of PP2C phosphatase activity function as abscisic acid sensors. Science *324*, 1064–1068.
9. Nishimura, N., Sarkeshik, A., Nito, K., Park, S.-Y., Wang, A., Carvalho, P.C., Lee, S., Caddell, D.F., Cutler, S.R., Chory, J., et al. (2010). PYR/PYL/RCAR family members are major in-vivo ABI1 protein phosphatase 2C-interacting proteins in Arabidopsis. Plant J. *61*, 290–299.
10. Hu, H., Boisson-Dernier, A., Israelsson-Nordström, M., Böhmer, M., Xue, S., Ries, A., Godoski, J., Kuhn, J.M., and Schroeder, J.I. (2010). Carbonic anhydrases are upstream regulators of CO_2-controlled stomatal movements in guard cells. Nat. Cell Biol. *12*, 87–93, 1–18.
11. Hashimoto, M., Negi, J., Young, J., Israelsson, M., Schroeder, J.I., and Iba, K. (2006). Arabidopsis HT1 kinase controls stomatal movements in response to CO_2. Nat. Cell Biol. *8*, 391–397.
12. Marten, H., Hyun, T., Gomi, K., Seo, S., Hedrich, R., and Roelfsema, M.R.G. (2008). Silencing of NtMPK4 impairs CO-induced stomatal closure, activation of anion channels and cytosolic Casignals in Nicotiana tabacum guard cells. Plant J. *55*, 698–708.
13. Tian, W., Hou, C., Ren, Z., Pan, Y., Jia, J., Zhang, H., Bai, F., Zhang, P., Zhu, H., He, Y., et al. (2015). A molecular pathway for CO_2 response in Arabidopsis guard cells. Nat. Commun. *6*, 6057.

14. Hashimoto-Sugimoto, M., Higaki, T., Yaeno, T., Nagami, A., Irie, M., Fujimi, M., Miyamoto, M., Akita, K., Negi, J., Shirasu, K., et al. (2013). A Munc13-like protein in Arabidopsis mediates H+-ATPase translocation that is essential for stomatal responses. Nat. Commun. *4*, 2215.

15. Roelfsema, M.R.G., and Prins, H.B.A. (1995). Effect of abscisic acid on stomatal opening in isolated epidermal strips of *abi* mutants of *Arabidopsis thaliana*. Physiol. Plant. *95*, 373–378.

16. Webb, A.A., and Hetherington, A.M. (1997). Convergence of the abscisic acid, CO_2, and extracellular calcium signal transduction pathways in stomatal guard cells. Plant Physiol. *114*, 1557–1560.

17. Leymarie, J., Vavasseur, A., and Lascève, G. (1998). CO_2 sensing in stomata of *abi1-1* and *abi2-1* mutants of *Arabidopsis thaliana*. Plant Physiol. Biochem. *36*, 539–543.

18. Merilo, E., Laanemets, K., Hu, H., Xue, S., Jakobson, L., Tulva, I., Gonzalez-Guzman, M., Rodriguez, P.L., Schroeder, J.I., Broschè, M., and Kollist, H. (2013). PYR/RCAR receptors contribute to ozone-, reduced air humidity-, darkness-, and CO_2-induced stomatal regulation. Plant Physiol. *162*, 1652–1668.

19. Webb, A.A.R., McAinsh, M.R., Mansfield, T.A., and Hetherington, A.M. (1996). Carbon dioxide induces increases in guard cell cytosolic free calcium. Plant J. *9*, 297–304.

20. Young, J.J., Mehta, S., Israelsson, M., Godoski, J., Grill, E., and Schroeder, J.I. (2006). $CO_{(2)}$ signaling in guard cells: calcium sensitivity response modulation, a $Ca^{(2+)}$-independent phase, and $CO_{(2)}$ insensitivity of the *gca2* mutant. Proc. Natl. Acad. Sci. USA *103*, 7506–7511.

21. Xue, S., Hu, H., Ries, A., Merilo, E., Kollist, H., and Schroeder, J.I. (2011). Central functions of bicarbonate in S-type anion channel activation and OST1 protein kinase in CO_2 signal transduction in guard cell. EMBO J. *30*, 1645–1658.

22. Negi, J., Matsuda, O., Nagasawa, T., Oba, Y., Takahashi, H., Kawai-Yamada, M., Uchimiya, H., Hashimoto, M., and Iba, K. (2008). CO_2 regulator SLAC1 and its homologues are essential for anion homeostasis in plant cells. Nature *452*, 483–486.

23. Vahisalu, T., Kollist, H., Wang, Y.-F., Nishimura, N., Chan, W.-Y., Valerio, G., Lamminmäki, A., Brosché, M., Moldau, H., Desikan, R., et al. (2008). SLAC1 is required for plant guard cell S-type anion channel function in stomatal signalling. Nature *452*, 487–491.

24. Meyer, S., Mumm, P., Imes, D., Endler, A., Weder, B., Al-Rasheid, K.A.S., Geiger, D., Marten, I., Martinoia, E., and Hedrich, R. (2010). *AtALMT12* represents an R-type anion channel required for stomatal movement in Arabidopsis guard cells. Plant J. *63*, 1054–1062.

25. Woodward, F.I., and Kelly, C.K. (1995). The influence of CO_2 concentration on stomatal density. New Phytol. *131*, 311–327.

26. Woodward, F.I. (1987). Stomatal numbers are sensitive to increases in CO_2 from pre-industrial levels. Nat. *327*, 617–618.

27. Tanaka, Y., Nose, T., Jikumaru, Y., and Kamiya, Y. (2013). ABA inhibits entry into stomatal-lineage development in Arabidopsis leaves. Plant J. *74*, 448–457.

28. Lau, O.S., and Bergmann, D.C. (2012). Stomatal development: a plant's perspective on cell polarity, cell fate transitions and intercellular communication. Development *139*, 3683–3692.

29. Pillitteri, L.J., and Torii, K.U. (2012). Mechanisms of stomatal development. Annu. Rev. Plant Biol. *63*, 591–614.

30. Casson, S.A., and Hetherington, A.M. (2010). Environmental regulation of stomatal development. Curr. Opin. Plant Biol. *13*, 90–95.

31. Gray, J.E., Holroyd, G.H., van der Lee, F.M., Bahrami, A.R., Sijmons, P.C., Woodward, F.I., Schuch, W., and Hetherington, A.M. (2000). The HIC signalling pathway links CO_2 perception to stomatal development. Nature *408*, 713–716.

32. Engineer, C.B., Ghassemian, M., Anderson, J.C., Peck, S.C., Hu, H., and Schroeder, J.I. (2014). Carbonic anhydrases, EPF2 and a novel protease mediate CO_2 control of stomatal development. Nature *513*, 246–250.

33. Lake, J.A., and Woodward, F.I. (2008). Response of stomatal numbers to CO_2 and humidity: control by transpiration rate and abscisic acid. New Phytol. *179*, 397–404.

34. Chater, C.C.C., Oliver, J., Casson, S., and Gray, J.E. (2014). Putting the brakes on: abscisic acid as a central environmental regulator of stomatal development. New Phytol. *202*, 376–391.

35. Kwak, J.M., Mori, I.C., Pei, Z.M., Leonhardt, N., Torres, M.A., Dangl, J.L., Bloom, R.E., Bodde, S., Jones, J.D.G., and Schroeder, J.I. (2003). NADPH oxidase *AtrbohD* and *AtrbohF* genes function in ROS-dependent ABA signaling in Arabidopsis. EMBO J. *22*, 2623–2633.

36. Pei, Z.-M., Murata, Y., Benning, G., Thomine, S., Klüsener, B., Allen, G.J., Grill, E., and Schroeder, J.I. (2000). Calcium channels activated by hydrogen peroxide mediate abscisic acid signalling in guard cells. Nature *406*, 731–734.

37. Zhang, X., Zhang, L., Dong, F., Gao, J., Galbraith, D.W., and Song, C.-P. (2001). Hydrogen peroxide is involved in abscisic acid-induced stomatal closure in *Vicia faba*. Plant Physiol. *126*, 1438–1448.

38. Kolla, V.A., Vavasseur, A., and Raghavendra, A.S. (2007). Hydrogen peroxide production is an early event during bicarbonate induced stomatal closure in abaxial epidermis of Arabidopsis. Planta *225*, 1421–1429.

39. Gonzalez-Guzman, M., Pizzio, G.A., Antoni, R., Vera-Sirera, F., Merilo, E., Bassel, G.W., Fernández, M.A., Holdsworth, M.J., Perez-Amador, M.A., Kollist, H., and Rodriguez, P.L. (2012). Arabidopsis PYR/PYL/RCAR receptors play a major role in quantitative regulation of stomatal aperture and transcriptional response to abscisic acid. Plant Cell *24*, 2483–2496.

40. Scott, I., and Logan, D.C. (2008). Mitochondria and cell death pathways in plants: Actions speak louder than words. Plant Signal. Behav. *3*, 475–477.

41. Mori, I.C., Pinontoan, R., Kawano, T., and Muto, S. (2001). Involvement of superoxide generation in salicylic acid-induced stomatal closure in *Vicia faba*. Plant Cell Physiol. *42*, 1383–1388.

42. Fahnenstich, H., Scarpeci, T.E., Valle, E.M., Flügge, U.-I., and Maurino, V.G. (2008). Generation of hydrogen peroxide in chloroplasts of Arabidopsis overexpressing glycolate oxidase as an inducible system to study oxidative stress. Plant Physiol. *148*, 719–729.

43. Park, S.-Y., Fung, P., Nishimura, N., Jensen, D.R., Fujii, H., Zhao, Y., Lumba, S., Santiago, J., Rodrigues, A., Chow, T.F., et al. (2009). Abscisic acid inhibits type 2C protein phosphatases via the PYR/PYL family of START proteins. Science *324*, 1068–1071.

44. Nishimura, N., Hitomi, K., Arvai, A.S., Rambo, R.P., Hitomi, C., Cutler, S.R., Schroeder, J.I., and Getzoff, E.D. (2009). Structural mechanism of abscisic acid binding and signaling by dimeric PYR1. Science *326*, 1373–1379.

45. Cutler, S.R., Rodriguez, P.L., Finkelstein, R.R., and Abrams, S.R. (2010). Abscisic acid: emergence of a core signaling network. Annu. Rev. Plant Biol. *61*, 651–679.

46. Koornneef, M., Jorna, M.L., Brinkhorst-van der Swan, D.L.C., and Karssen, C.M. (1982). The isolation of abscisic acid (ABA) deficient mutants by selection of induced revertants in non-germinating gibberellin sensitive lines of *Arabidopsis thaliana* (L.) heynh. Theor. Appl. Genet. *61*, 385–393.

47. Léon-Kloosterziel, K.M., Gil, M.A., Ruijs, G.J., Jacobsen, S.E., Olszewski, N.E., Schwartz, S.H., Zeevaart, J.A.D., and Koornneef, M. (1996). Isolation and characterization of abscisic acid-deficient Arabidopsis mutants at two new loci. Plant J. *10*, 655–661.

48. Frey, A., Effroy, D., Lefebvre, V., Seo, M., Perreau, F., Berger, A., Sechet, J., To, A., North, H.M., and Marion-Poll, A. (2012). Epoxycarotenoid cleavage by NCED5 fine-tunes ABA accumulation and affects seed dormancy and drought tolerance with other NCED family members. Plant J. *70*, 501–512.

49. Bauer, H., Ache, P., Lautner, S., Fromm, J., Hartung, W., Al-Rasheid, K.A., Sonnewald, S., Sonnewald, U., Kneitz, S., Lachmann, N., et al. (2013). The stomatal response to reduced relative humidity requires guard cell-autonomous ABA synthesis. Curr. Biol. *23*, 53–57.

50. Shimizu, T., Miyakawa, S., Esaki, T., Mizuno, H., Masujima, T., Koshiba, T., and Seo, M. (2015). Live single-cell plant hormone analysis by video-mass spectrometry. Plant Cell Physiol. *56*, 1287–1296.

51. Waadt, R., Hitomi, K., Nishimura, N., Hitomi, C., Adams, S.R., Getzoff, E.D., and Schroeder, J.I. (2014). FRET-based reporters for the direct visualization of abscisic acid concentration changes and distribution in Arabidopsis. eLife *3*, e01739.

52. Jones, A.M., Danielson, J.Å.H., Manojkumar, S.N., Lanquar, V., Grossmann, G., and Frommer, W.B. (2014). Abscisic acid dynamics in roots detected with genetically encoded FRET sensors. eLife *3*, e01741.

53. MacAlister, C.A., Ohashi-Ito, K., and Bergmann, D.C. (2007). Transcription factor control of asymmetric cell divisions that establish the stomatal lineage. Nature *445*, 537–540.

54. Hunt, L., and Gray, J.E. (2009). The signaling peptide EPF2 controls asymmetric cell divisions during stomatal development. Curr. Biol. *19*, 864–869.

55. Piñero, M.C., Houdusse, F., Garcia-Mina, J.M., Garnica, M., and Del Amor, F.M. (2014). Regulation of hormonal responses of sweet pepper as affected by salinity and elevated CO_2 concentration. Physiol. Plant. *151*, 375–389.

56. Murata, Y., Mori, I.C., and Munemasa, S. (2015). Diverse stomatal signaling and the signal integration mechanism. Annu. Rev. Plant Biol. *66*, 369–392.

57. Pantin, F., Renaud, J., Barbier, F., Vavasseur, A., Le Thiec, D., Rose, C., Bariac, T., Casson, S., McLachlan, D.H., Hetherington, A.M., et al. (2013). Developmental priming of stomatal sensitivity to abscisic acid by leaf microclimate. Curr. Biol. *23*, 1805–1811.

58. Ruszala, E.M., Beerling, D.J., Franks, P.J., Chater, C., Casson, S.A., Gray, J.E., and Hetherington, A.M. (2011). Land plants acquired active stomatal control early in their evolutionary history. Curr. Biol. *21*, 1030–1035.

59. Chater, C., Kamisugi, Y., Movahedi, M., Fleming, A., Cuming, A.C., Gray, J.E., and Beerling, D.J. (2011). Regulatory mechanism controlling stomatal behavior conserved across 400 million years of land plant evolution. Curr. Biol. *21*, 1025–1029.

60. Hauser, F., Waadt, R., and Schroeder, J.I. (2011). Evolution of abscisic acid synthesis and signaling mechanisms. Curr. Biol. *21*, R346–R355.

Current Biology

Supplemental Information

Elevated CO_2-Induced Responses in Stomata Require ABA and ABA Signaling

Caspar Chater, Kai Peng, Mahsa Movahedi, Jessica A. Dunn, Heather J. Walker, Yun-Kuan Liang, Deirdre H. McLachlan, Stuart Casson, Jean Charles Isner, Ian Wilson, Steven J. Neill, Rainer Hedrich, Julie E. Gray, and Alistair M. Hetherington

Supplemental Figure S1, related to Figure 4B.

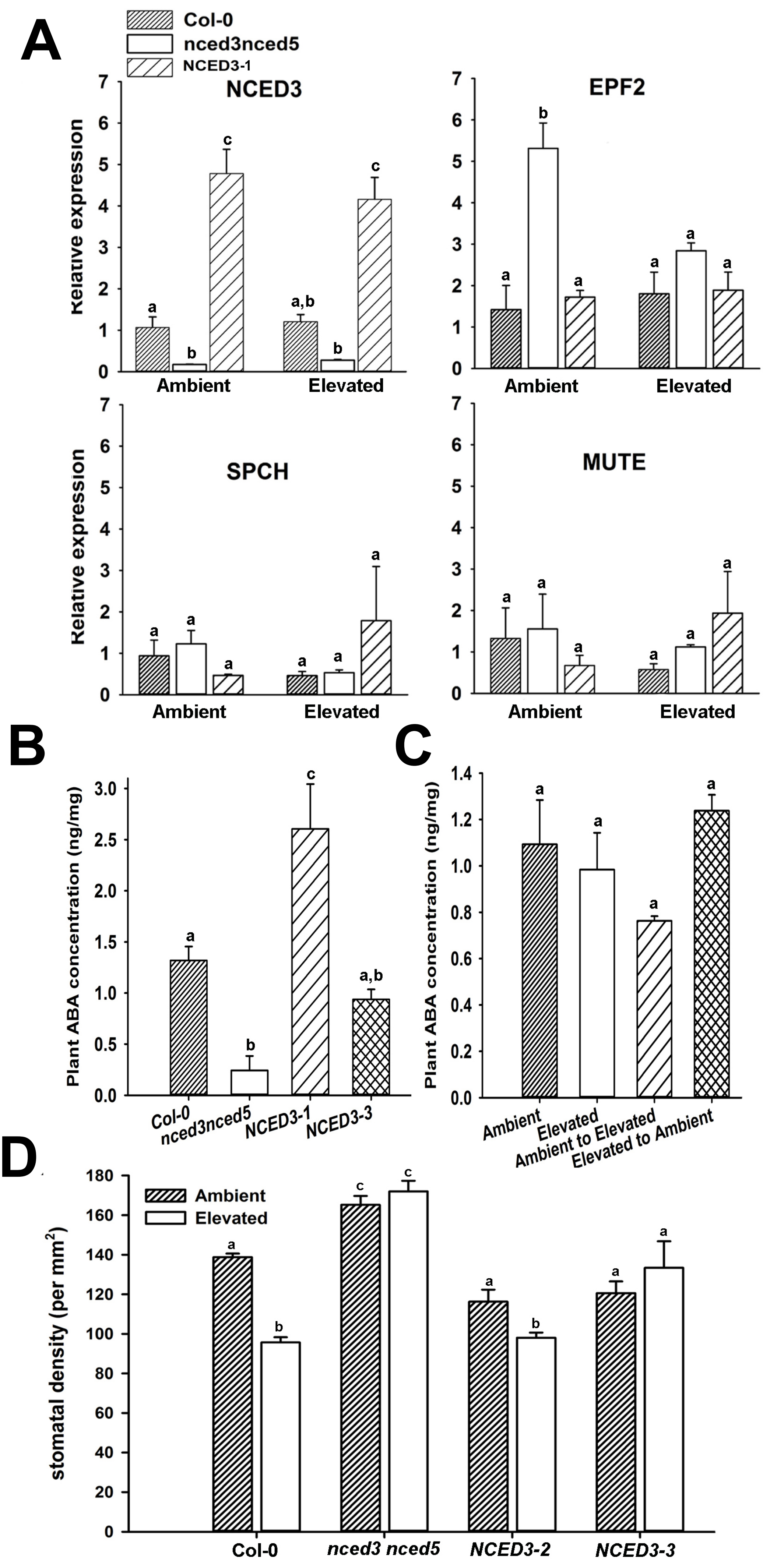

Supplemental Figure S2, related to Figure 4B and Figure S1.

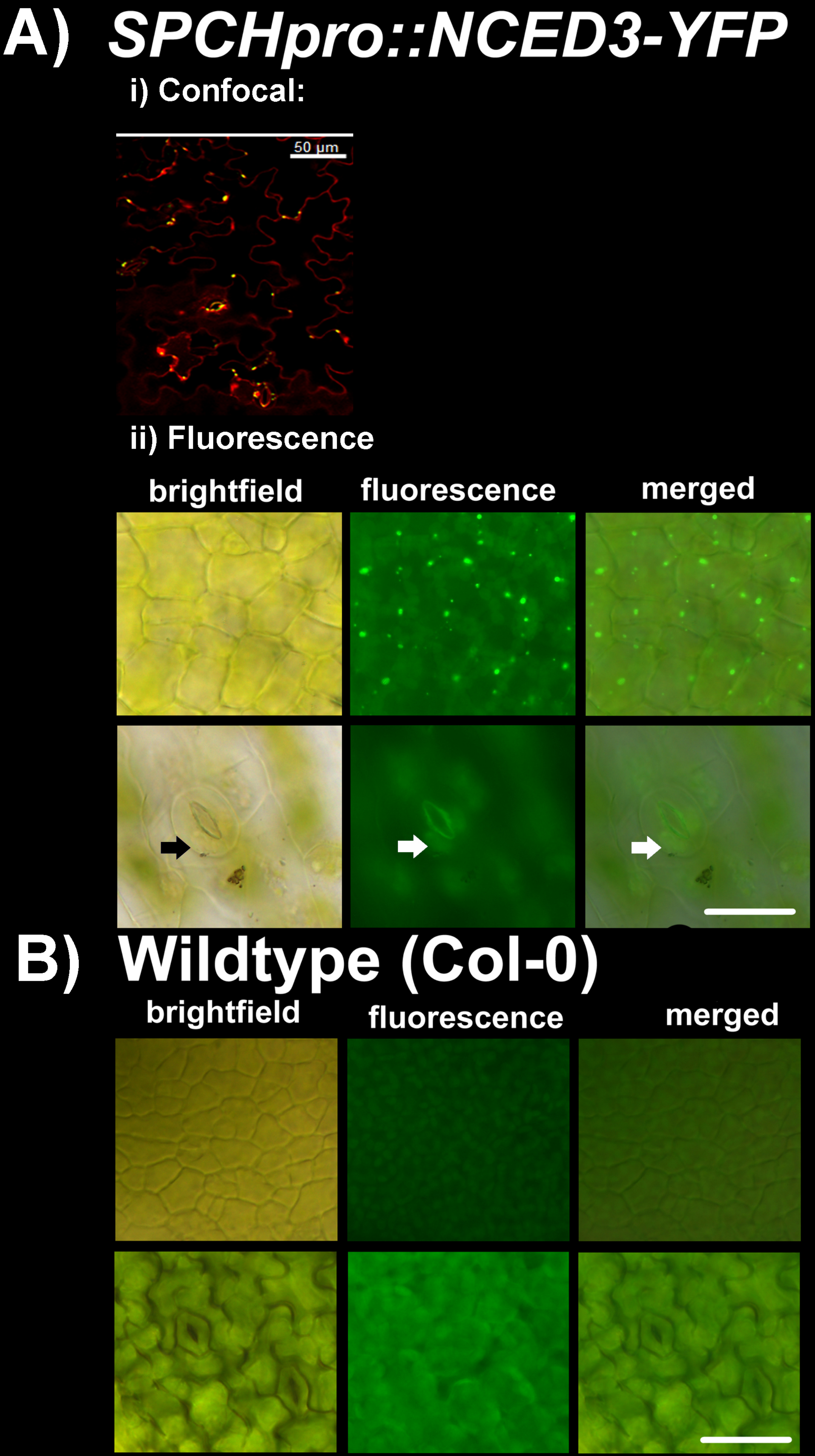

Supplemental Figure Legends

Supplemental Figure S1, related to Figure 4B.

A. qRT-PCR of *NCED3* transcripts showed substantially reduced levels in *nced3 nced5* (ambient[CO_2], ANOVA, p <0.05) which was restored to higher levels in the *SPCHpro::NCED3-YFP* line NCED3-1. Expression of EPF2 was significantly increased in *nced3 nced5* compared with wild-type (ambient [CO_2], ANOVA, p = 0.0016). Expression levels of SPEECHLESS and MUTE were also analysed.
B. ABA levels were significantly reduced in the *nced3 nced5* line compared to wild-type (ANOVA, p = 0.0349) and restored to higher levels in *SPCHpro::NCED3-YFP* lines NCED3-1 and NCED3-3.
C. Levels of ABA measured from wild-type seedlings across [CO_2] growth conditions and reciprocal transfer experiments were not significantly different to each other (ANOVA, p > 0.05).
D. .Stomatal densities of two additional independently transformed lines of *$SPCH_{pro}$::NCED3-YFP*, NCED3-2 and NCED3-3, were not significantly different to wild-type when grown under ambient [CO_2] and density of one (*$SPCH_{pro}$::NCED3*) was significantly reduced when grown at elevated [CO_2] (ANOVA, P <0.001).

Supplemental Figure S2, related to Figure 4B and Figure S1.

A. Fluorescence is detected in the developing epidermis of ten day old *SPCHpro::NCED3-YFP* expressing seedlings in a punctate pattern consistent with localisation to plastids as previously shown by immunolocalization [S1], where the NCED3 step of ABA biosynthesis occurs. (i) Confocal micrograph, with propidium iodide cell wall marker (red), and YFP signal (yellow) (scale = 50 µm), and (ii) brightfield and fluorescence microscopy with overlay (scale = 20 µm). Arrows: a lower level of fluorescence can be observed in chloroplasts of some of the young guard cells in the developing epidermis.
B. YFP fluorescence is not present in wild-type seedling epidermis, shown by brightfield and fluorescence microscopy with overlay (scale = 20 µm).

Supplemental Experimental Procedures

Plant material and growth conditions

Arabidopsis thaliana ecotype Col-0 was used in this investigation. Seeds of *pyr1,pyl1,pyl4* and *pyr1,pyl1,pyl2,pyl4* in the Col-0 background were obtained from Dr Sean Cutler (University of California, Riverside, USA)*, aba3-1*, and *aba3-1::MYB60$_{pro}$::ABA3,* and *nced3 nced5* mutants have been previously described [S2, S3]. For the experiments reported in Figures 1A & B, seeds were surface-sterilised, rinsed, and sown onto a peat based mixed compost of Sinclair multipurpose compost and horticultural silver sand (William Sinclair Horticultural, UK) in a 3:1 ratio in plug-trays. After stratification (4°C in the dark, 2 days), trays were transferred into a Microclima growth cabinet (Snijders Scientific, Netherlands) with a 22°C (day)/ 20°C (night) temperature cycle; 10/14hr light/dark cycle; 70% relative humidity, PPD 120 µmol $m^{-2}s^{-1}$, at ambient [CO_2], approx. 400ppm. 14 day old plants were re-potted into 5 cm Arabaskets in 51-cell plug trays (Beta Tech, Gent, Belgium). For the experiments reported in Figures 1C, D & F, 2, 3, and 4, and Supplemental Figure S1 seeds were sown onto M3 and perlite in a 4:1 ratio in plug-trays and transferred into a growth cabinet (Conviron) with growth conditions 22°C (day)/ 16°C (night) temperature cycle; 9/15hr light/dark cycle; 70% relative humidity, PPD 150 µmol $m^{-2}s^{-1}$, at ambient [CO_2] (approx. 400ppm).

Measurement of stomatal aperture responses

Abaxial epidermis was removed from the youngest, fully expanded leaves of 5 to 6 week old plants and floated, cuticle-side up, on CO_2-free 10 mM MES/KOH (pH 6.15) in 5cm Petri dishes (Sterilin, UK) at 22°C. Epidermal peels were transferred to fresh Petri dishes and incubated for 2h in the light under a PPD of 150 µmol $m^{-2}s^{-1}$ in 50mM KCl, 10 mM MES/KOH (pH 6.15) at 22°C whilst being aerated with CO_2-free air by bubbling through the buffer solution. This treatment brought about stomatal opening. Peels were then either aerated with lab air (ambient, approx. 400 ppm [CO_2]- as measured by IRGA), or elevated [CO_2] from a pressurised cylinder containing CO_2 in air (BOC, Special Gasses, UK) by bubbling directly into the buffer. After 2h peels were removed, mounted on slides and measurements of stomatal aperture recorded using an inverted microscope (Leica DM-IRB, Leica UK) or a microscope (Olympus BX51), fitted camera (Olympus DP70), and ImageJ software v. 1.43u.

Forty stomatal pores were measured per treatment in three separate replicated experiments (total stomatal number = 120; n = 3). To avoid experimenter bias, measurements were performed without the researcher being aware of the sample identity. Additional treatments were as follows: The ROS scavengers Tempol (4-hydroxy-2,2,6,6-tetramethylpiperydine-1-oxyl) (Sigma-Aldrich, UK) and Tiron (4,5-dihydroxy-1,3-benzenedisulfonic acid) (Sigma-Aldrich, UK) were dissolved in water and used at a final concentration of 10 mM [S4] and added immediately prior to the addition of elevated CO_2. Data were analysed using ANOVA (MINITAB and Sigmaplot 12).

ROS and viability assays

To assess viability following the treatments described above, epidermal peels were stained with 0.01 % FDA (Fluorescein Diacetate) (Sigma-Aldrich, UK) from 10 % (w/v) stock solution in ethanol (final ethanol concentration 0.1 % (v/v)) for 10 minutes in dark. Peels were washed in 50mM KCl, 10 mM MES/KOH (pH 6.15) at 22°C for 10 min in the light (PPD 150 µmol $m^{-2}s^{-1}$) and the mean fluorescence intensity (total fluorescence of one guard cell / total area of guard cell) was measured on a Zeiss Axiovert 200M microscope with XBO 75 fluorescent lamp and a GFP filter set fitted with a CCD camera (Hamamatsu) controlled by the Volocity software (Version 5, Improvision). Data were analysed using ANOVA (MINITAB and Sigmaplot 12).

To estimate ROS generation abaxial epidermal peels were prepared as described above and incubated in 50mM KCl, 10 mM MES/KOH (pH 6.15) at 22°C, PPD of 100 µmol $m^{-2}s^{-1}$ whilst being aerated with CO_2-free air for 2h. They were then transferred to 50mM KCl, 10 mM MES/KOH (pH 6.15) at 22°C , PPD of 100 µmol $m^{-2}s^{-1}$ and either aerated with ambient [CO_2] or elevated [CO_2] for 2h. The epidermal peels were then loaded (by pipetting) with 25 µM (final concentration) H_2DCF-DA (2',7'-dichlorodihydrofluorescein diacetate) (Invitrogen, UK) from a 25 mM stock in DMSO for 10 minutes in dark. They were then washed in 50mM KCl, 10 mM MES/KOH (pH 6.15) at 22°C for 10 min in the light (PPD 150 µmol $m^{-2}s^{-1}$) to remove excess H_2DCF-DA and the fluorescence intensity was measured as using an Olympus BX51 fluorescence microscope and ImageJ as described in [S5]. For ROS analysis, pixel intensities of forty stomatal areas (fluorescence zone of two guard cells) relative to their background intensities (four equivalent sized areas surrounding each stoma) were measured per

treatment in three separate replicated experiments (total stomatal number = 120; n = 3). Fluorescence intensities were normalised to those of controls.

Stomatal density measurements

Dental resin (Coltene Whaledent, Switzerland) was applied to the abaxial surfaces of fully expanded leaves and nail varnish peels were taken from set resin. Cell counts were taken from four fields of view from the widest area of four leaves each from at least five plants of each genotype from both growth conditions. Data were analysed using ANOVA (MINITAB and Sigmaplot 12).

Generation of *$SPCH_{pro}$::NCED3* Plants

The Arabidopsis *NCED3* gene was PCR amplified using a 5' primer with overhanging ApaI restriction site (5'- GAGAT TGG GCC CAT GGC TTC TTT CAC GGC A-3') and a 3' primer with overhanging XhoI restriction site (5'- AAA CTC GAG CAC GAC CTG CTT CGC CAA -3'). The *pGKGWY::proSPCH::PHYB* vector [S6] was digested with ApaI and XhoI to remove the *PHYB* gene, and the *NCED3* PCR product was digested with ApaI and XhoI ligated with the *pGKGWY::proSPCH::* backbone to obtain the plasmid *pGKGWY::proSPCH::NCED3* and sequenced. Arabidopsis *nced3 nced5* mutant plants were transformed by vacuum infiltration with *Agrobacterium tumefaciens*, selected for kanamycin resistance. PCR-based confirmation of the stably inserted *proSPCH*::*NCED3-YFP* construct, and RT-PCR and qRT-PCR were carried out to determine *NCED3* expression. F2 generation were used for stomatal analysis.

Gas exchange measurements

Stomatal conductance was measured using infrared gas analysis. Measurements were performed using a portable photosynthesis system attached to a leaf chamber fluorometer with a 2cm^2 leaf area (LI-COR 6400-40). CO_2 was scrubbed from external air using soda lime and resupplied from a liquid CO_2 cartridge (Liss), to maintain CO_2 concentrations of either 500 or 1000ppm. Relative humidity in the chamber was maintained between 60-75% using self-indicating desiccant (Drierite). Air flow was 200µmol s^{-1}, light intensity was 1000µmol.m^{-2}.s^{-1} and the chamber temperature was controlled at 20°C. For each measurement, an individual mature leaf was placed in the leaf chamber, while still attached to the plant.

Photographs of each leaf in the chamber gasket were taken and leaf area used for measuring gas exchange was calculated from these using ImageJ. Leaves were left in the chamber for one hour before measurements were taken, in order to allow them to acclimatise to chamber conditions and for gas exchange to stabilise. Measurements were then logged every 30 seconds for 10 minutes and mean stomatal conductance values taken from these.

Gene Expression

For quantitative RT-PCR analysis (qPCR), RNA was extracted from 2 week old seedlings using the Quick-RNA™ MiniPrep (Zymo Research) plant RNA extraction kit with on-column DNase treatment according to the manufacturer's instructions. RNA was reverse transcribed with High Capacity Reverse Transcriptase (Applied Biosystems). Transcript abundance of target genes was assayed using SYBR Green/JumpStart Taq ReadyMix qPCR Master Mix (Sigma Aldrich). The *ACTIN2* and *UBC21* genes were used as controls, as transcript levels remained constant under all treatments and relative expression levels were calculated using the ΔΔCt method. Expression was calculated relative to that of Col-0 seedlings grown at 400 ppm [CO_2]. Three biological repeats and three technical repeats were performed for each sample and used to calculate s.e.m. values. Reaction conditions were (1 x 95°C - 10 mins; 40 x 95°C - 15s/57°C – 20s/72°C – 30s). Details of primer sequences can be found in supplemental experimental procedures.

**Primer**	**Forward (5'-3')**<br>**Reverse (5'-3')**
SPCH	AACGGTGTCGCATAAGATCC<br>CAAGAGCCAAATCTTCAAGAGC
NCED3	AAAGCCATCGGTGAGCTTCA<br>GCAGCTCTGGCGTAGAATAGC
ACTIN2	TCAGATGCCCAGAAGTGTGTT<br>CCGTACAGATCCTTCCTGATAT
UBC21	GAATGCTTGGAGTCCTGCTTG<br>CTCAGGATGAGCCATCAATGC
EPF2	CCAACATCCTCCCATCCAAGTC<br>TGAGCAATCTGGCAACCTAGACC
MUTE	AACGTCGAAAGACCCTAAACCG<br>TTAGCATGAGGGGAGTTACAGC

ABA quantification by Mass Spectrometry

100 mg of above-ground plant tissue was ground to a powder in liquid nitrogen and homogenised in cold water:chloroform:methanol extraction matrix (80: 200: 470 µl) before vortexing and resting for 30 minutes. 400 µl of cold H_2O was then added before centrifugation at 14000 rpm for 2 minutes. The aqueous phase was transferred to a new tube before an additional 400 µl of cold sterile water was added to re-extract from the chloroform layer. Both aqueous phases were combined, and frozen prior to analysis by mass spectrometry.

ABA concentration was analysed using UPLC-MS Acquity coupled to Synapt G2 mass spectrometer (Waters, UK). Data was collected in negative mode at capillary voltage 2.5 kV, source temperature 120 $^{\circ}$C, desolvation temperature 350 $^{\circ}$C, sample cone 20V, extraction cone 4V, desolvation gas flow 900L hr^{-1}, cone gas flow 20L hr^{-1}.UPLC separation was obtained using a linear gradient system (0 minutes 5% acetonitrile in H_20, 3 minutes 35% acetonitrile, 6 minutes 100% acetonitrile, 7.5 minutes 100% acetonitrile, 7.6 minutes 5% acetonitrile) and an Acquity UPLC Peptide BEH C18 column (size= 2.1 mm x 50mm, pore size= 130Å, particle size= 1.7µm) with a flow rate of 0.6 ml min^{-1} and an injection volume of 10µl. Peak size was compared to (+)- ABA standard (Sigma Aldrich).

Confocal microscopy

Confocal images were captured with a Leica TCS SP5 confocal microscope after counterstaining tissues with 10 µg ml^{-1} propidium iodide.

Supplemental References

S1. Endo, A., Sawada, Y., Takahashi, H., Okamoto, M., Ikegami, K., Koiwai, H., Seo, M., Toyomasu, T., Mitsuhashi, W., Shinozaki, K., et al. (2008). Drought Induction of Arabidopsis 9-cis-Epoxycarotenoid Dioxygenase Occurs in Vascular Parenchyma Cells. Plant Phy. *147*, 1984-1993.

S2. Frey, A., Effroy, D., Lefebvre, V., Seo, M., Perreau, F., Berger, A., Sechet, J., To, A., North, H.M., and Marion-Poll, A. (2012). Epoxycarotenoid cleavage by NCED5 fine-tunes ABA accumulation and affects seed dormancy and drought tolerance with other NCED family members. The Plant J. *70*, 501-512.

S3. Bauer, H., Ache, P., Lautner, S., Fromm, J., Hartung, W., Al-Rasheid, Khaled A.S., Sonnewald, S., Sonnewald, U., Kneitz, S., Lachmann, N., et al. (2013). The Stomatal Response to Reduced Relative Humidity Requires Guard Cell-Autonomous ABA Synthesis. Curr. Biol. *23*, 53-57.

S4. Yamada, J., Yoshimura, S., Yamakawa, H., Sawada, M., Nakagawa, M., Hara, S., Kaku, Y., Iwama, T., Naganawa, T., Banno, Y., et al. (2003). Cell permeable ROS scavengers, Tiron and Tempol, rescue PC12 cell death caused by pyrogallol or hypoxia/reoxygenation. Neurosci. Res. *45*, 1-8.

S5. Gavet, O., and Pines, J. (2010). Progressive Activation of CyclinB1-Cdk1 Coordinates Entry to Mitosis. Dev. Cell *18*, 533-543.

S6. Casson, S.A., and Hetherington, A.M. (2014). Phytochrome B Is Required for Light-Mediated Systemic Control of Stomatal Development. Curr. Biol. *24*, 1216-1221.
